# Multi-Analytical Techniques for the Study of Burial Clothes of Polish King Sigismund III Vasa (1566–1633) and His Wife Constance Habsburg (1588–1631)

**DOI:** 10.3390/molecules29010192

**Published:** 2023-12-28

**Authors:** Magdalena Śliwka-Kaszyńska, Maria Cybulska, Anna Drążkowska, Sławomir Kuberski, Jakub Karczewski, Anna Marzec, Przemysław Rybiński

**Affiliations:** 1 Department of Organic Chemistry, Faculty of Chemistry, Gdansk University of Technology, 80-233 Gdańsk, Poland; 2 Faculty of Material Technologies and Textile Design, Institute of Architecture of Textiles, Lodz University of Technology, 90-924 Lodz, Poland; maria.cybulska@p.lodz.pl; 3 Faculty of History, Institute of Archaeology, Nicolaus Copernicus University in Torun, 87-100 Torun, Poland; annadr@umk.pl; 4 Faculty of Process and Environmental Engineering, Lodz University of Technology, 93-005 Lodz, Poland; slawomir.kuberski@p.lodz.pl; 5 Faculty of Applied Physics and Mathematics, Gdansk University of Technology, 80-233 Gdańsk, Poland; jakkarcz@pg.edu.pl; 6 Faculty of Chemistry, Lodz University of Technology, 90-924 Lodz, Poland; anna.marzec@p.lodz.pl; 7 Institute of Chemistry, Jan Kochanowski University, 25-369 Kielce, Poland; przemyslaw.rybinski@ujk.edu.pl

**Keywords:** grave robes, natural dyes, mordants, metal threads, tandem mass spectrometry, scanning electron microscopy, energy-dispersive X-ray spectroscopy, thermogravimetric analysis

## Abstract

The subjects of this research are the burial clothes of Polish King Sigismund III Vasa and his wife Constance, which were woven and embroidered with silk and metal threads. Fragments of the textiles underwent spectroscopic, spectrometric, and thermogravimetric analyses. The hydrofluoric acid extraction method was improved to isolate various classes of dyes from the textile samples that had direct contact with human remains. High-performance liquid chromatography, coupled with diode array and tandem mass spectrometry detectors with electrospray ionization (HPLC-DAD-ESI-MS/MS) facilitated the detection and identification of colorants present in the textiles. Cochineal, indigo-, madder-, orchil-, and tannin-producing plants were identified as the sources of dyes used. Scanning electron microscopy with an energy-dispersive X-ray detector (SEM-EDS) was employed to identify and characterize the silk fibers and mordants and the metal threads. The presence of iron, aluminum, sodium, and calcium in the silk threads suggests their potential use as mordants. The analysis of the metal threads revealed that most of them were made from flattened gilded silver wire, with only a few being cut from a sheet of metal. Typical degradation mechanisms of metal threads were shown, resulting from both burial environment and earlier manufacturing process, and the use of the textiles in clothing, i.e., a significant loss of the gold layer was observed in most of silver gilt threads, caused by abrasion and delamination. The results of the thermal analysis confirmed the presence of silk and silver threads in the examined textiles.

## 1. Introduction

The study of archaeological textiles has rapidly evolved into a burgeoning field of research, yielding crucial insights into ancient dyeing and weaving techniques. The identification of both the organic and inorganic components of historical textiles is essential for their comprehensive characterization and for assessing the technologies used in their production. To analyze historical samples, researchers employ a multitude of analytical methods and procedures aimed at extracting data on both their organic and inorganic elements. Due to the typically limited availability of samples taken from historical objects, the identification of organic and inorganic matter requires the use of sensitive and selective analytical methods.

In recent years, several methods have been developed for the analysis of natural dyes in historical textiles. The most common technique used for the identification is high-performance liquid chromatography coupled with spectrophotometric detection (HPLC-UV-Vis) [1,2,3], and tandem mass spectrometric detection with electrospray ionization (HPLC-ESI-MS) [4,5,6,7,8,9,10,11,12,13,14] or atmospheric pressure chemical ionization (HPLC-APCI-MS) [15,16]. Some examples of the application of matrix-assisted laser desorption/ionization time-of-flight mass spectrometry (MALDI-TOF-MS) imaging to study archeological textiles has been also described [17,18,19]. The application of high-resolution mass spectrometry (HRMS) and tandem mass spectrometry (MS/MS) has unveiled new possibilities for elucidating not only the molecular structures of dyes, but also identifying their degradation products [13,20,21,22,23,24,25,26,27,28,29].

For many years, examining dyestuff components was often hindered by the need for unacceptably large sample sizes to isolate dyes from different matrices before analysis. Nowadays, thanks to advancements in analytical methods, the determination of dyes from historical samples has become feasible. However, even with current sophisticated analytical methods, the sampling may not always be viable, particularly when dealing with valuable and fragile fabrics of great historical significance. To address this issue, modern non-invasive surface methods, including UV-Vis reflectance, luminescence spectroscopy [30,31,32,33], and multispectral imaging (MSI) [4,24,25,34,35] have emerged as valuable techniques capable of examining dye distribution on surfaces and discerning specific dyes. However, when it concerns yellow dyes, non-invasive examinations yield limited data since many of yellows exhibit similar fluorescence and reflectance spectra, making them indistinguishable from one another [4,36].

Other types of non-destructive methods, including scanning electron microscopy (SEM) with energy dispersive X-ray spectroscopy (EDX) and energy dispersive spectroscopy (EDS), X-ray fluorescence spectroscopy (XRF), and Particle-induced X-ray spectroscopy (PIXE), are employed to examine not only the surface morphology and elemental composition of inorganic samples but also to identify the types and extent of corrosion products present on the surface [37,38,39,40]. SEM-EDS is an especially productive method for investigating metal threads from different periods and cultures. It is often used to assess the structural features and metal composition, characterize the substrate metal, and analyze surface layers such as gilding [41,42,43,44,45].

The royal crypts at the Archcathedral Basilica of Saint Stanislaus and Saint Wenceslaus on the Wawel Hill hold immense historical and cultural significance in Poland. They are the final resting place of Polish kings, as well as numerous bishops, political leaders, and poets. In one of the crypts the remains of the Polish King Sigismund III Vasa and his wife, Queen Constance Habsburg, were placed in tin sarcophagi. Although the royal sarcophagi have been periodically opened throughout the centuries, the clothing of the royal couple and the fabrics from which they were crafted have never been subject to examination. Consequently, the research undertaken in this article holds immense importance as it provides a rare opportunity to explore and analyze the burial robes of the King and Queen of Poland, shedding light on their construction and materials.

In this study, twenty-two textile fragments of clothes found in the royal crypts were examined. Scanning electron microscopy was employed for fiber characterization. An analysis of the metal and the metal-wrapped threads was conducted using scanning electron microscopy with an energy-dispersive X-ray detector (SEM-EDS). Mordant ions were also identified by SEM-EDS. The extraction procedure based on hydrofluoric acid was utilized to isolate dyes from the burial attire. The dye analysis was performed through liquid chromatography with UV-Vis and mass spectrometric detection with atmospheric pressure electrospray ionization in negative and positive modes (LC-DAD-ESI-MS). A thermogravimetric analysis (TGA) was employed to evaluate and characterize the metal threads and fibers used in the manufacture of historical textiles.

## 2. Results and Discussion

### 2.1. Origin of Textile Samples

**Figure 1:**
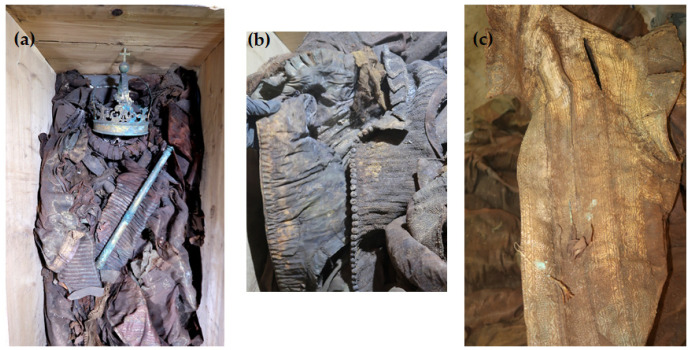
Images of the burial cloths found in the royal crypts: (**a**) remains and robes of Queen Constance in a temporary coffin, (**b**) the waistcoat, (**c**) fragment of the outer gown; photo by W. Głowa.

In 2018, the Vasa crypt was opened in connection with the planned conservation work on the tin sarcophagi of King Sigismund III Vasa and his wife, Queen Constance Habsburg. During the transfer of the remains to the temporary coffin, for the first time an opportunity arose to take samples of textiles to examine the burial robes of the royal couple and the fabrics from which they were sewn (Figure 1).

The robes were found to be heavily soiled, covered with dust and organic impurities. Every item of clothing was badly crumpled and discolored to a great extent. Many of the fabrics exhibited extensive dark stains, particularly on the clothing covering the back part of the bodies. In both burials, the garments displayed heavy discoloration with shades of brown, tea, gold, and occasionally hints of green and red. The remains of Queen Constance, although they were very damaged, lay in an anatomical arrangement. The queen was dressed in clothing made of silk and metal threads, including an outer gown (a robe), a waistcoat, and a very wide skirt. The coffin contained also a pillow. The remains of King Sigismund III Vasa were much more damaged and displaced. They were not anatomically arranged, and some of them were even falling apart. The garments were mixed up and the whole burial had clear signs of plundering. The king’s coffin contained the following garments: a royal robe (a cope), dalmatic, alb, cassock, doublet, gloves, stockings, and shoes. Twenty-two textile samples from different parts of the burial clothes were gathered for a comprehensive investigation (see Table 1 for the object description).

### 2.2. Microscopic and Spectroscopic Studies

#### 2.2.1. SEM-EDS Analysis of Metal Threads

Metal threads have been used in textiles since ancient times in embroidery and weaving. Their purpose was to emphasize the status of the people wearing them, their power and wealth in the case of rulers and aristocracy [46,47,48].

Metal threads can have various forms. Initially, they were metal strips cut from a beaten sheet of metal. Flat metal thread, known also as a lamella, made by this method is referred as “beaten and cut” [45,46,48]. Such a thread was applied directly or was used to create a compound thread by wrapping a strip around the fibrous core and creating a wrapped metal thread [49]. In the Middle Ages, when the production of wires improved, they also began to be used in the production of silk fabrics and haberdashery, mainly as weft threads. Soon, a new method of producing flat metal thread was developed, which involved drawing, rolling, and flattening the metal filament “drawn and rolled”. This method became the main method of producing lamella, but in Italy, where the textiles in question probably come from, the earlier method “beaten and cut” was still used in the 16th and 17th centuries [46,48].

Metal threads were made of various metals and their alloys, mainly of gold, silver and copper. To produce gold thread, alloys with silver and copper were used, the content of which could change the shade of gold. In the 16th and 17th centuries, where the analyzed textiles come from, silver-plated and/or gilded copper threads were also used [44,50,51].

**Figure 2:**
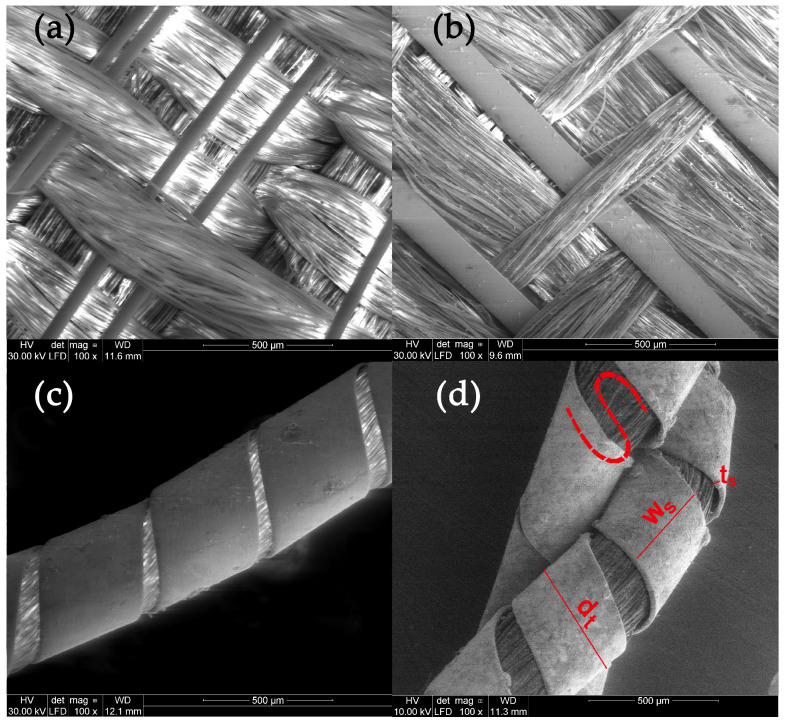
Types of the metal threads: (**a**) metal filament, (**b**) flat metal thread, (**c**) metal wrapped thread, (**d**) doubled metal-wrapped thread and parameters of the thread structure.

Scanning electron microscopy (SEM), and energy dispersive X-ray spectroscopy (EDS) are valuable tools that provide insights into both the physical and chemical characteristics of fibers. The threads in the analyzed fabrics have three forms: metal filaments, i.e., wire threads, flat metal thread called lamella, and metal-wrapped thread, in which the fibrous core is wrapped with a metal strip. The last one was also doubled (Figure 2).

**Figure 3:**
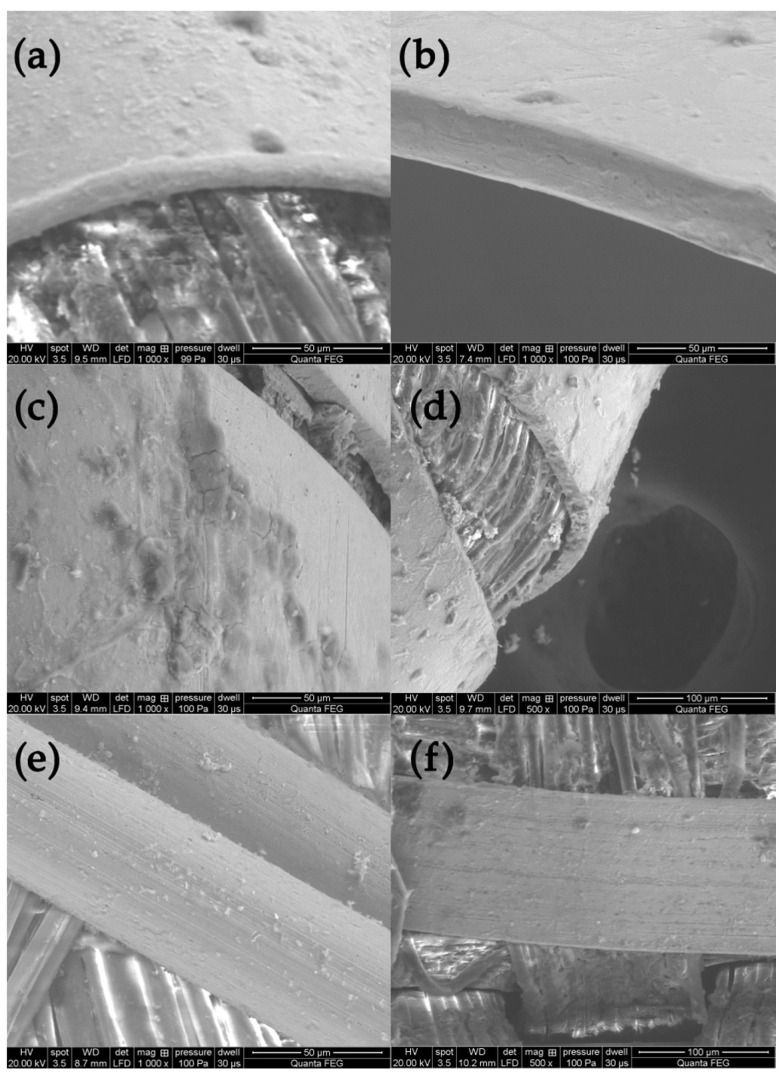
Metal threads—characteristics of manufacturing methods: (**a**,**b**) rounded edges of the thread made of flatten wire, (**c**,**d**) sharp edges of threads cut form the metal sheet, (**e**,**f**) traces of drawing the wire.

On the basis of the SEM images, the structural parameters of the threads were determined: the diameter in the case of a metal filament, width (w_s_) and thickness (t_s_) of the lamella for a flat and braided metal thread, diameter of the thread (d_t_), including the metal filament. For metal-wrapped threads, the number of warps per unit length and wrapping direction, similarly to the twist of textile yarns, described by the letters S or Z, (Figure 2d) were also determined. A series of elemental composition measurements were also performed for each thread and average values of the percentage of three metals—gold, silver and copper—were determined. The test results are presented in Table 2. Most of the threads are gold-plated silver with a higher or lower copper content, which depends on the type and technique of the thread manufacturing. Filaments (wires) used as threads in silks imported from Italy have the lowest copper content. The fabric Sig8 from which the royal cope was sewn is Italian, with the brocading and patterning wefts made of gilded almost pure silver. The high copper content mainly concerns decorative tapes used to trim the garments, as well as threads in the striped silk of the lining of Queen Constance’s outer robe Con15. Based on the SEM images, it was possible to determine which method was used to produce individual metal threads. Threads made of flattened wire bear traces of drawing, visible in the form of stripes running along the thread. This is particularly visible on the surface of metal filaments, but also, despite rolling, on the surface of a flat metal thread made of wire (Figure 3e,f).

Another characteristic feature of “drawn and rolled” threads are rounded edges, which results from the manufacturing technique itself (Figure 3a,b). In contrast, threads cut from a metal sheet have sharp edges with visible cutting marks (Figure 3c,d).

Most, as much as 75%, of the tested threads were made of flattened wire, which confirms the widespread use of this method in 16th and 17th century. Metal threads from the royal robes are characterized by a large variety in terms of structural parameters. The diameter of the metal filaments ranged from 54 to 66 μm.

The width of the flat metal thread is much more diverse, from 122 to 653 μm; the widest and thickest were used in haberdashery products, while the thinnest in woven silks, with the exception of the silver gilt boucle weft in the Con22 upholstery fabric. These parameters do not depend on the method of making the thread. The threads are characterized by greater or lesser coverage of the surface with a metal strip. The low number of wrappings coupled with the low diameter of the lamella show that the wrapping is very sparse and the fibrous core of the metal-wrapped thread is visible, as in the case of weft threads in samples Con15 (brocading weft) and Con16 (weft) (see photographs in Table 1). However, this is not a saving of expensive material, but rather a solution often found in fashionable fabrics from this period [52].

**Figure 4:**
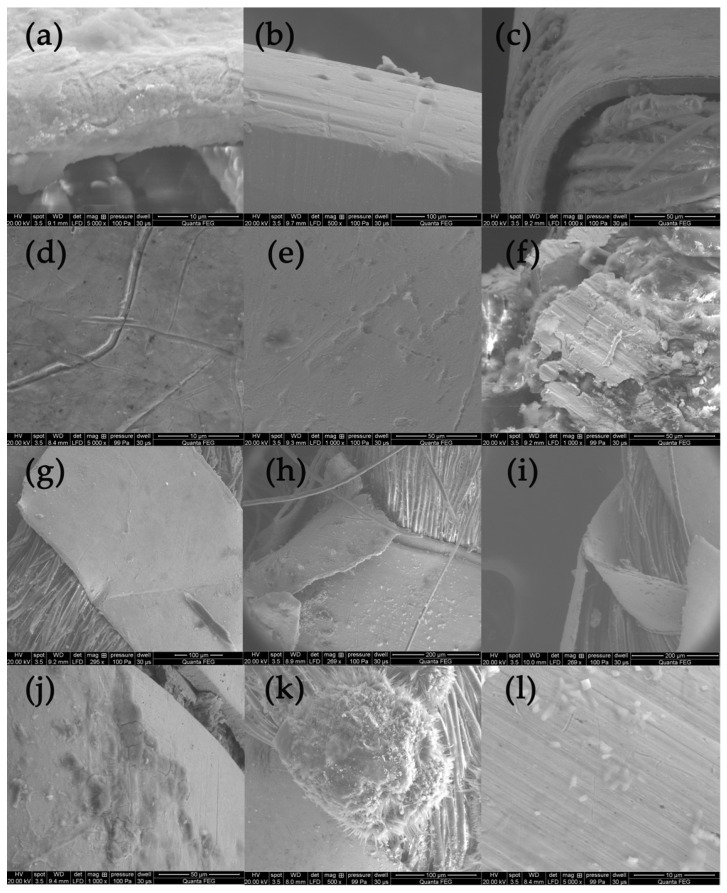
SEM images presenting destruction of metal threads: (**a**–**c**) loss of gold layer; (**d**) scratches, (**e,f**) corrosion, (**g**–**i**) defragmentation, (**j**–**l**) crystalline structures.

The analysis of the state of preservation of the metal threads was based on the analysis of SEM images and elemental composition (Table S1 and Figure S1). Figure 4 shows SEM images of fragments of metal threads with different types of destruction: the loss of the gold layer due to abrasion or delamination (Figure 4a–c); mechanical damage of surface (scratches) that could have occurred both in the weaving process and in the use of the fabric (Figure 4d); the corrosion of metal due to the influence of the environment (Figure 4e,f); the defragmentation of the metal wrapping (Figure 4g–i); and formation of crystalline structures on the metal surface or in its vicinity (Figure 4j–l). Silver gilt thread (sample Sig4) was made using the “beaten and cut” method, so the gold layer was only on the outside of the strip.

Due to biodegradation processes, the inner side was corroded and the corrosion products formed a kind of pseudomorph, reflecting the fibers of the silk core inside the wrapping (Figure 4f) [53]. The analysis of the threads revealed that the degree of destruction of metal threads is influenced not only by the archaeological environment but also by the quality of the threads themselves and how they are used in the final product. When the threads form a compact, dense structure, it becomes much more difficult for harmful environmental factors to access them. Among the tested threads, the greatest damage occurred in areas where they form loose structures, such as single loose threads in tassels and loops in woven textiles (see photographs in Table 1 and Figure S2) [54]. A good example of the impact of both the manufacturing technique and the textile structure is a fabric with bouclé effects created using silver and silver-gilt wefts (Con22). Before being inserted into the fabric structure, the metal threads of the weft were twisted and wrapped around a thin metal rod, which were then inserted together into the shed. After closing the shed, the rod was pulled out, and the weft formed loops over the ground fabric (Figure S2a,b). As a result, the threads are more vulnerable to damage, not only due to exposure to harmful factors in the archaeological environment but also because of mechanical stress and susceptibility to abrasion.

#### 2.2.2. SEM-EDS Analysis of Silk Threads

**Figure 5:**
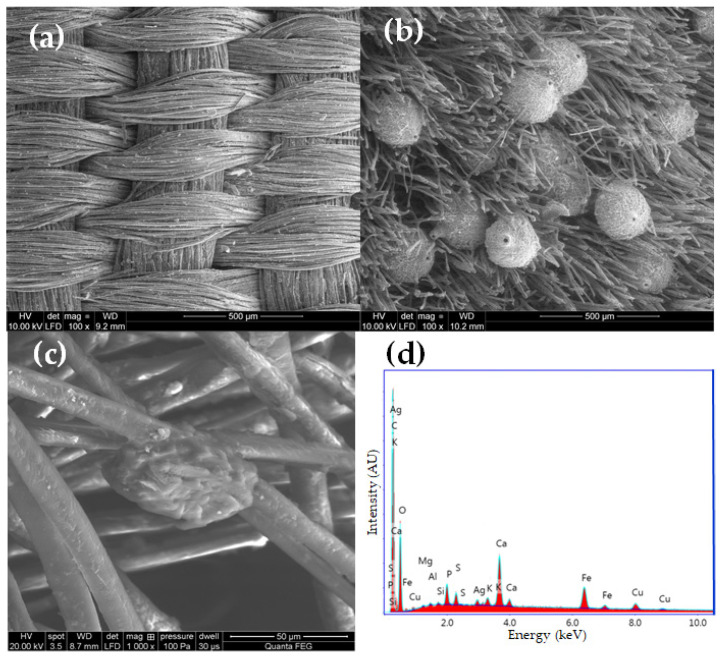
SEM micrographs of (**a**) textile Sig2 (mag. 100×), (**b**) textile Sig9 (mag. 100×), (**c**) textile Sig5 (mag. 1000×), (**d**) SEM-EDS spectrum of textile Sig2.

Visual information, combined with the chemical properties of the fiber, frequently proves sufficient for identification purposes. All the fabrics found in royal crypt were made of silk. Historical testimonies about the King’s death and funeral have survived, as well as merchant archives about silk fabrics purchased for the funeral’s needs. These historical sources were confirmed by the SEM analysis. Natural fibers have significantly different structures and surface characteristics. Long smooth, fine filaments with a diameter of 5–10 mm, visible in the SEM images, clearly allow us to identify the fibers as silk (Figure 5a–c).

The textiles remained in relatively good condition after over 300 years in the environment of the crypts. Their structural integrity remains intact, and defects such as holes, tears, and other damage are relatively rare. The robes worn by the royal couple were soiled with surfaces covered with dust and organic impurities. Most samples were contaminated with inorganic particles. Micrographs of the historical textiles also show biological colonization (Figure 5b).

Similarly to metal threads, silk fibers suffered the most damage in objects with a relatively loose structure, such as fringes, loops, and pile, making them vulnerable to the penetration and growth of microorganisms. In the case of silk fabrics, velvet pile exhibited the most significant biodegradation, while the ground fabric, also made of silk, remained nearly untouched (Sig9).

Mordant dyes need the use of coordination metals to fix the dyestuff molecules to textile fibers which may influence its final hue [55,56]. For example, madder exhibits violet tinting when paired with iron, red with aluminum, and pink with tin mordants. To determine the presence of inorganic elements used as mordants and potential contaminants originating from the crypts, EDS analyses were conducted (Table 3). Figure 5d provides a representative EDS spectrum of a silk fiber sample.

The investigated samples did not show significant differences in elemental composition, although the relative amounts of elements varied. In many samples, the presence of calcium (Ca), iron (Fe), aluminum (Al), potassium (K), and traces of copper (Cu) was observed. Aluminum, iron, and potassium are likely associated with mordants necessary for achieving durable colors. Calcium, primarily in the form of calcium carbonate, was commonly added to the dye bath to deepen or enhance the shades. Copper was found in seven samples, though it remains unclear whether it was intentionally added as a mordant agent or if it resulted from metal thread residues. Notably, iron and copper salts are known to darken red and yellow mordant dyes, thereby influencing the final fabric color, while aluminum salts do not alter the hue of dyed fabrics. Additionally, few samples contained trace amounts of sodium (Na), magnesium (Mg), silicon (Si), and phosphorus (P), which could be attributed to the utilization of textiles throughout their lifespan or remnants from adorned coffins that were painted or upholstered with fabrics. Several elements were consistently detected, including carbon and oxygen, originating from silk proteins. The presence of sulfur was unsurprising, as this element is naturally found in animal fibers.

### 2.3. Thermogravimetric Analysis of Threads

Thermal analysis techniques, such as thermogravimetric analyses (TGAs), are very useful for evaluating and characterizing metal threads and fibers used in the manufacture of historical textiles. The thermal decompositions of the threads are affected by the fiber type, metal components, experimental conditions (our experiments were mainly carried out in a N_2_ or air atmosphere), and metal corrosion processes [57].

**Figure 6:**
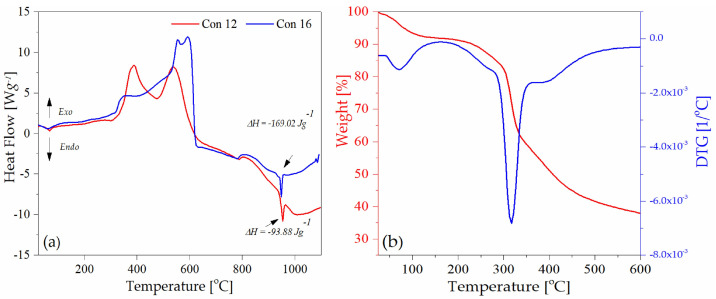
Thermograms of samples: (**a**) Con12 and Con16, DSC; (**b**) Sig11, TGA-DTG.

Table 2 displays the elemental composition of metal threads, including silver, copper, and gold, identified through SEM-EDS in the studied threads. The TGA-DSC technique was used for selected samples Con12, Con13, Con16, Con17, and Sig7 as a complementary test to the SEM-EDS method. In all samples examined by the thermal analysis (Con12, Con13, Con16, Con17, and Sig7), the presence of silver was detected (Figure S4a–e). This metal is responsible for the endothermic peak around 954 °C in the DSC curve (Figure 6a; the melting point of silver is at 962 °C). Among the considered samples, the greatest enthalpy value (−169.02 J·g^−1^) was observed for a metal thread (specimen Con12), which is consistent with the SEM-EDS analysis and confirms the highest silver content in the considered sample.

The thermogram of the sample Sig11 (satin fabric) demonstrated two distinct zones of mass loss (Figure 6b), which are characteristic for silk fibers. The first endothermic peak was formed at around 75 °C, indicating the presence of water. After the dehydration process, a clear mass loss is only visible between 290 and 350 °C with a peak maximum at 324 °C in the DTG curve, which can be ascribed to the breakdown of side groups of an amino acid residue as well as the cleavage of the peptide bonds in silk fibers [58].

### 2.4. Identification of the Colorants (HPLC-DAD-ESI-MS)

Archaeological fabrics, especially burial textiles, are more susceptible to damage and decay than other artifacts. Due to taphonomic processes their colors usually fade and change, primarily turning yellowish brown over extended periods of time. For this reason, it is challenging to discern the original color, tint, or hue of the dyed fabric [14,59]. All textiles analyzed in this study have undergone numerous alterations over the years, modifying their original hue and shade. The variations in shade, saturation, and shine of the fabric can be attributed to the degradation of silk fibers, as well as the degradation of some colorants [60], making their identification problematic. Furthermore, as the layers of fabric encasing the deceased directly interact with bodily fluids and underwent biodegradation in the course of taphonomic processes, stains may develop and permeate adjacent materials. As a consequence, dyes from other fabrics may be detected on fabrics that were not originally dyed with these substances.

For the LC-MS examination, only silk fragments of textile samples without metal threads were selected. To isolate dyes from the textile samples, a mild extraction method with hydrofluoric acid was used. The extracts’ components were separated using high-performance liquid chromatography on a C-18 reversed-phase column. The eluted compounds were characterized with diode array detector and mass spectrometer connected in-line, including their retention times, UV-vis spectra, and mass spectra. A mass spectrometer with atmospheric pressure electrospray ionization was operated in both negative and positive modes (HPLC-ESI(−/+)-MS). LC-MS was also employed to analyze reference dyestuff samples in extracts from madder (*Rubia tinctorum* L.), cochineal (*Dactylopius coccus* Costa), oak gallnuts, and indigo (*Isatis tinctoria*) in order to provide further identification.

**Figure 7:**
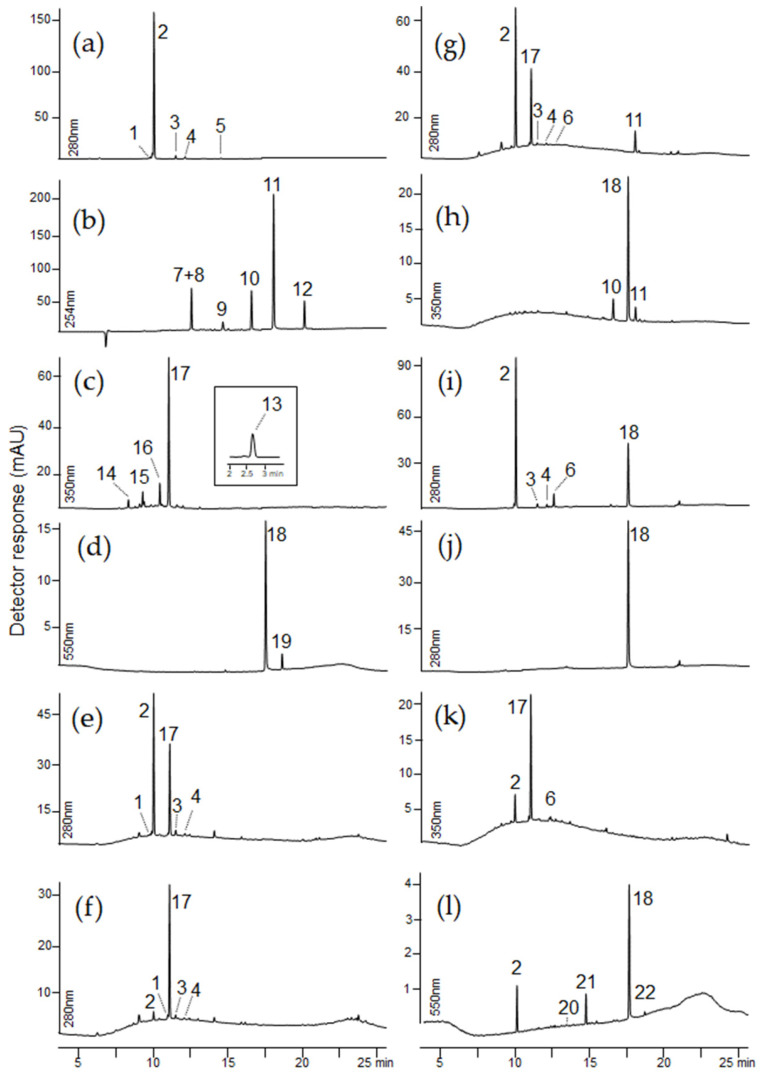
Chromatograms of extracts taken from samples: (**a**) cochineal, (**b**) madder, (**c**) gallnuts, (**d**) indigo, (**e**) Sig2, (**f**) Sig3, (**g**) Sig11, (**h**) Con15, (**i**) Con17, (**j**) Con20, (**k**) Con22, (**l**) Con14. For chromatographic conditions, see experimental section.

Figure 7 shows chromatograms of selected textile extracts and dyeing raw materials. A summary of the identified colorants and possible dyestuffs sources are presented in Table 3. The retention times, molecular ions, main fragment ions, and proposed identification of the detected dyes are summarized in Table 4.

An oil painting from 1632 by a court painter Christian Melich, “Sigismund III Vasa on catafalque”, depicts Sigismund III Vasa on his funeral bed in an official bright/white robes adorned with gold and red patterned fabric (see Figure S3). However, it is uncertain whether this depiction accurately reflects the funeral robes of the King or if it is more of an artistic interpretation by the painter.

The crypt containing the king’s remains has been plundered many times over the centuries, but the entire set of his funeral clothes has been preserved to this day. A very limited number of coloring substances were detected in the king’s burial clothes. Red and brown dyestuffs were found in the majority of the textiles examined. The LC-MS analysis revealed the presence of carminic acid in nine out of eleven royal robes. Carminic acid ([M−H]^−^ at *m*/*z* 491) loses carbon dioxide forming fragment ion at *m*/*z* 447. The *C*-glucoside moiety of this molecule decomposes via cross-ring cleavages (losses of 90, 120, and 148 Da) resulting in formation of ions at *m*/*z* 357 [^0,3^X−H−CO_2_]^−^, 327 [^0,2^X−H−CO_2_]^−^, and 299 [^0,1^X−H−CO_2_]^−^. Carminic acid is the primary dye ingredient derived from several species of Porphyrophora, e.g., Armenian cochineal (*Porphyrophora hameli* Brandt), Polish cochineal (*Porphyrophora polonica* L.), and also from American cochineal (*Dactylopius coccus* Costa). A significant amount of carminic acid was found in textiles taken from the doublet (samples Sig2 and Sig3), cassock (sample Sig11), and coffin upholstery (Sig9). Only trace amounts of this red dyestuff were detected in the fabrics from the dalmatic (samples Sig4, Sig5, Sig6, Sig7) and the alb (Sig10). Smaller amounts of flavokermesic acid 2-*C*-glucoside (dcII; *m*/*z* 475), kermesic acid 7-*C*-glucofuranoside (dcIV; *m*/*z* 491), and kermesic acid 7-*C*-glucofuranoside (dcVII; *m*/*z* 491) were also detected in textile samples labeled as Sig2, Sig3, and Sig11. DcIV and dcVII differ from carminic acid only in a sugar moiety. Their MS spectra are similar and the main losses observed are typical of *C*-glycosides of carboxylic acids. In the spectrum of dcII ([M−H]^−^ at *m*/*z* 475), a series of signals were observed: [M−H−CO_2_]^−^ at *m*/*z* 431, [^0,3^X−H−CO_2_]^−^ at *m*/*z* 341, [^0,3^X−H−H_2_O−CO_2_]^−^ at *m*/*z* 323, [^0,2^X−H−CO_2_]^−^ at *m*/*z* 311, and [^0,1^X−H−CO_2_]^−∙^ at *m*/*z* 282. These compounds are minor colorants present both in American and Polish cochineals [6,7,9]. In the 16th century, European sources of cochineal were supplanted by the American variety of cochineal, which had previously been skillfully domesticated and cultivated by the indigenous peoples of the New World. Subsequently, it was commercialized by the Spanish empire [55,61]. Since all of the above-mentioned dyeing raw materials contain carminic acid as the main ingredient, it is difficult to determine which of them was used for dyeing. However, historical documents and preserved recipes from the 17th century suggest that American cochineal, most likely *Dactylopius coccus* Costa, was used to dye the fabrics of the royal robes [55,56].

**Figure 8:**
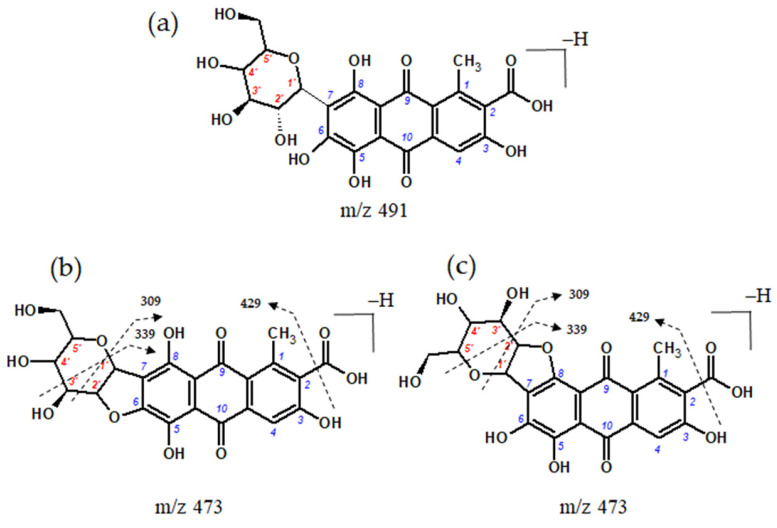
Chemical structure of (**a**) carminic acid, (**b**,**c**) dehydrated forms of carminic acid, with the numeration of carbon atoms within aglycone (blue) and sugar moiety (red), and proposed fragmentation directions.

In the extract of textile sample Sig11, a compound with a molecular anion at *m*/*z* 473 was also detected. This molecular anion differs by 18 atomic mass units from the ion of carminic acid. Therefore, it may have originated from carminic acid through the elimination of water, involving the hydroxyl substituent at position C-2′ of the sugar moiety and the hydroxyl groups at either the C-6 or C-8 position of the aglycone [6,62]. This transformation is facilitated by the hydrogen bond between the ether oxygen atom of the sugar ring and the 6- or 8-hydroxyl group (Figure 8).

We have previously identified this compound in burial textiles excavated from 17th-century crypts in the Basilica of St. Francis of Assisi in Cracow [14] on the basis of the ESI(−)-QTOF mass spectrum with pseudo-molecular ion [M−H]^−^ at *m*/*z* 473.0727, corresponding to the elemental composition of C_22_H_17_O_12_ (mass difference 0.3 ppm). Dehydrated carminic acid forms a fragment ion [M−H−CO_2_]^−^ at *m*/*z* 429. The formation of a dihydrofuran moiety leads to changes in the further fragmentation of the sugar molecule leading to ions at *m*/*z* 309 of the [^0,2^X−H−CO_2_]^−^ (loss of 120 Da) and at *m*/*z* 339 ([^0,3^X−H−CO_2_]^−^, loss of 90 Da). The presence of the dehydrated form of carminic acid in royal burial gowns suggests that this compound may be a marker for the transformation of carminic acid resulting from taphonomic processes because it is not found in any cochineal species or textiles dyed using this raw material.

The red anthraquinone purpurin ([M−H]^−^ at *m*/*z* 255) was found in the textile sample Sig11, in addition to the previously mentioned dyes. Purpurin ([M−H]^−^ at *m*/*z* 255) was identified based on the presence of characteristic fragment ions formed by the further loss of CO and CO_2_, or both these molecules together: [M−H−CO]^−^ at *m*/*z* 227, [M−H−CO−CO_2_]^−^ at *m*/*z* 183, and [M−H−3CO]^−^ at *m*/*z* 171. Identification of this compound was straightforward as a purpurin standard is available. Purpurin as a red anthraquinone dye is found in the roots of different species of the *Rubiaceae* family, including madder (*Rubia tinctorum* L.), wild madder (*Rubia peregrina* L.), munjeet (*Rubia cordifolia* L.), and several species of *Relbunium* native to South America. Among these, madder played a particularly significant role as a red dye. While it originated in India, it was extensively cultivated in Europe and the Middle East during the high-demand period. Having less tinctorial power compared to cochineal, madder offered the advantage of producing a wide range of shades, including pinks, purples, reds, and even blacks when used with different mordants [56]. The presence of purpurin in the textile sample Sig11, along with the absence of alizarin and other anthraquinone dyes, makes it impossible to determine which species from the *Rubiaceae* family was used in the dyeing process of this royal fabric. However, historical literature sources mention the use of a mixture of madder and cochineal to create a brilliant red known as “demi-grain” [55].

Ellagic acid, sourced from tannin-producing plants, was found in most of the burial fabrics of the King. This phenolic compound exhibited the [M−H]^−^ and [2M−H]^−^ ions at *m*/*z* 301 and 603, respectively. The mass spectrum of the deprotonated molecule exhibited the following fragmentation pattern: *m*/*z* 229 (loss of CO_2_ and CO) and *m*/*z* 185 (loss of two CO_2_ and one CO). Ellagic acid alters the final shade of the textiles, primarily by darkening the original color, but also adds weight to the silk fibers lost during the degumming process [22,55]. The process of weighting, wherein silk is treated with a finishing substance such as galls, increases weight, adds density, and improves draping quality.

In sample Sig1, which was taken from the lining of the robe, ellagic acid was detected as the sole dye. The cope (Sig8) probably was not pigmented because not even a trace amount of dye was found in it.

Based on the obtained results, it can be assumed that some of the clothes in which King Sigismund was buried, such as the doublet, cassock, and dalmatic tassel, or their fragments, were dyed red. Regarding fabric fragments from the dalmatic and alb, it is uncertain whether the presence of trace amounts of carminic acid results from cochineal used to dye them or if this dye seeped through from the adjacent layers of red clothes. This uncertainty may suggest that these robes were originally white, as depicted in the painting of King Sigismund on his deathbed.

The burial of Queen Constance has survived to our times in better condition than King Sigismund’s one. The exhumed remains of the Queen suggest that she was buried in an outfit composed of multiple layers of clothing adorned with numerous embroideries and richly decorated with metal threads, including an outer gown (robe), a waistcoat, and a very wide skirt. Based on the LC-MS analysis, the purple-colored fabric of the outer dress (sample Con14) was dyed with a cocktail of three different ingredients, including extracts of cochineal, indigo, and orchil.

The major components in the extract of this fabric were indigotin and carminic acid (Figure 7l). Indigotin, with a pseudo-molecular ion [M−H]^−^ at *m*/*z* 261, has three less intensive fragment ions at *m*/*z* 233 [M−H−CO]^−^, 217 [M−CONH_2_]^−^, and 175 [M−H−CO−C_2_H_2_O]^−^.

Indigo could have been produced through a fermentation process using either European or Asian plants. Woad (*Isatis tinctoria* L.) is a native European plant, which has been in use on the Old Continent since ancient times. Following the discovery of the sea route to India, indigo from *Indigofera tinctoria* L. was imported to Europe, but its usage was relatively uncommon during that period. Towards the end of the 16th century, an increasing quantity of Asian indigo began arriving in Europe [55,56]. Initially, it was combined with woad, but by the 17th century, it had largely replaced woad-based indigo. Consequently, fabrics of Queen attire could be dyed using both *Indigofera* or *Isatis* species.

The chromatographic profile of Con14 extract indicated three minor components eluting at 14.3, 15.0, and 19.1 min, respectively. Based on their pseudomolecular ions [M+H]^+^ at *m*/*z* 362, *m*/*z* 363, and *m*/*z* 364, as well as fragmentation ions formed after the loss of small radicals such as methyl [M−H−CH_3_]^−•^ at *m*/*z* 347, 348, and 349; hydroxyl [M−H−OH]^−•^ at *m*/*z* 345, 346, and 347; and aminyl [M−H−NH_2_]^−•^ at *m*/*z* 346, 347, and 348 or small neutrals (H_2_O), these coloring substances were identified as α-aminoorceimine, α-aminoorcein, and α-hydroxyorcein [9].

These orchil dyes were obtained by fermentation of extracts from lichens of different species in the presence of ammonia and air to give various orcein derivatives. Orchil can be obtained from different lichen species, which are native to specific regions or geographical areas. The most important lichens were *Rocella tinctoria* D.C. and *Rocella fuciformis* D.C. which can be found not only in Europe but also in the East Indies, South and Central America, and the West Coast of Africa. One of the main uses of lichen dyes was to substitute for the noble and expensive Tyrian purple dye, hence they were often call “false shellfish purples” [63].

The patterned fabric lining of the outer robe contained alizarin and purpurin, which are red components found in madder, as well as indigotin, the major component of blue indigo (sample Con15). Alizarin was identified based on the molecular anion [M−H]^−^ at *m*/*z* 239 and the fragment ions at *m*/*z* 211 [M−H−CO]^−^, 183 [M−H−2CO]^−^, and 167 [M−H−CO−CO_2_]^−^.

Since a mixture of red and blue dyes, similar to the lichens extract, also produces a purple color, it is highly likely that the queen’s outer dress was originally purple. The presence of coloring substances found in cochineal and indigo was also identified in fragments of the skirt (sample Con17) and the waistcoat (sample Con18) (Figure 7i). The presence of carminic acid, the dehydrated form of carminic acid, dc IV, dcVII, and indigotin, which in turn is a mixture of blue and red dyes, was confirmed. Only indigotin was detected in the outer fabric of the vest (samples Con19 and Con20, Figure 7j). The extract of a textile sample from the pillowcase (sample Con22) contains a significant amount of ellagic acid as well as minor amounts of carminic acid and its dehydrated form (Figure 7k).

## 3. Materials and Methods

### 3.1. Chemicals

Methanol (MeOH) and acetonitrile (ACN), both HPLC grade, were purchased from Merck (Darmstadt, Germany). Hydrofluoric acid (HF, 48% in water) and formic acid (FA, 98–100%) were purchased from Sigma-Aldrich (Steinheim, Germany). Dimethyl sulfoxide (DMSO, ACS grade) was obtained from Merck KGaA (Darmstadt, Germany). RC-4 membrane filters (0.2 μm) were purchased from Sartorius Stedim Biotech GmbH (Goettingen, Germany). All aqueous solutions were prepared using Milli Q water (Merck, Darmstadt, Germany). Raw dyestuff materials including madder roots, cochineal, oak gallnuts and indigo (*Indigofera* L.) were obtained from Kremer Pigmente (Aichstetten, Germany) in dried form, and were homogenized prior to the extraction procedure.

### 3.2. Equipment

A scanning electron microscope (SEM) with a secondary electron detector operated in high vacuum mode at an accelerating voltage of 10–20 kV (FEI Quanta FEG 250, Thermo Fisher Scientific, Waltham, MA, USA) was used to examine the morphology of the samples. The elements were identified by energy dispersive spectroscopy (EDS) using an ApolloX SDD spectrometer (Ametek, Berwin, PA, USA) at an accelerating voltage of 20 kV. Chromatographic analysis was performed using an Agilent Liquid Chromatograph Series 1290 (Agilent Technology, Waldbronn, Germany) containing a high speed binary pump (G7120A), autosampler (G7167B), thermostated column compartment (G7116B), diode-array detector (G1315C), and triple quadrupole mass spectrometer with AJS electrospray ionization source (G6470B). The Agilent MassHunter software (B 06.01) controlled the chromatographic system.

Thermogravimetric analysis (TGA) in two different atmospheres was applied to monitor the thermal degradation process, which is related to mass loss as a function of rising temperature. Thermogravimetric measurements (TGA/DSC1 analyzer, Mettler Toledo, Greifensee, Switzerland) were taken at a linear heating rate of 10 °C min^−1^ over the temperature range from ambient temperature to 1100 °C, in an air or N_2_ flow.

### 3.3. Extraction Procedure

Dyes were isolated from the burial fabrics using 500 µL of a mixture containing 8 M HF/MeOH/ACN/DMSO (2:1:1:1, *v*/*v*). Textile samples (~1 cm^2^) were placed in an ultrasonic bath for 30 min (2 × 15 min). After this time, mixtures were centrifuged at 9000 rpm for 5 min to separate the particulate matter. The supernatants, after the addition of acetone (100 µL), were kept in a refrigerator for 24 h in order to precipitate protein remains. Then, solutions were filtered over a 0.2 μm RC-4 syringe filter.

### 3.4. LC-MS Analysis

The extracts (2 µL) were injected onto RP-C_18_ silica gel column (Poroshell EC; 2.7 µm, 3.0 × 150 mm, Agilent Technologies, Waldbronn, Germany) thermostated at 40 °C. The mobile phase flow rate was 0.4 mL min^−1^, and elution was performed using 0.1% (*v*/*v*) formic acid in water (solvent A) and ACN/MeOH (1:1; *v*/*v*) (solvent B). Elution was performed with the following composition gradient: 0–2 min at 10% (B); 2–20 min 100% (B); 20–30 min 100% (B). The analysis was stopped after 30 min. The re-equilibration time of the column was 15 min at 10% (B). The UV signal was registered at 254, 280, 350, 550, and 600 nm. Mass-spectrometric data were recorded in negative and positive ionization scan modes (*m*/*z* 50–1000) in the following conditions: the nebulizer pressure, 45 psi; nitrogen flow rate, 5 L min^−1^; drying gas temperature, 300 °C; drying gas flow rate, 11 L min^−1^; and sheath gas temperature, 250 °C. The capillary voltage was 3.5 kV and the fragmentation voltages were 150 and 250 V.

## 4. Conclusions

Textile fragments of the funeral clothes of Polish King Sigismund III Vasa and his wife, Queen Constance, underwent spectroscopic, spectrometric, and thermogravimetric investigations.

Each of the techniques used allows us to obtain specific information about the structure of the materials from which the royal robes are made, their chemical composition, and state of preservation. Although each of them has its advantages and disadvantages, they are complementary and enabling us to obtain comprehensive knowledge about the analyzed textiles. Microscopic and spectroscopic techniques are non-invasive, while chromatographic and thermogravimetric analyses are invasive, leading to the irreversible destruction of the analyzed sample.

The visual and microscopic studies showed that most of the excavated textiles have preserved their integrity; however, they were heavily soiled and severely wrinkled and had undergone significant discoloration, particularly those on the back of the garments.

For the fiber identification and degradation assessment, SEM-EDS and TGA were employed. SEM micrographs of the silk fibers revealed smooth structures with longitudinal striations. The EDS analyses indicated the presence of various elements, including Ca, Fe, Al, K, and traces of Cu, along with carbon and oxygen derived from animal proteins. Elements like aluminum, iron, and potassium were likely associated with mordants used to achieve fast colors, while traces of copper, silica, and magnesium may have come from the utilization of textiles over a lifetime or from contaminants in the burial site.

The analyzed metal threads had three forms: metal filaments, i.e., wire; flat metal threads; and metal-wrapped threads, in which the fibrous core is wrapped with a metal strip. SEM-EDS showed that most of the threads are made of silver and silver gilt with a higher or lower copper content. Additionally, a significant loss of gold layer was observed in most of silver gilt threads, caused by abrasion and delamination.

HPLC-DAD-ESI-MS enabled the detection and identification of colorants present in the royal clothes. Several classes of dyes were isolated from textile samples using a mild hydrofluoric acid extraction method. Cochineal, indigo-, madder-, orchil-, and tannin-producing plants were identified as sources of dyes. Indigotin, carminic acid, ellagic acid dominated in the investigated burial textiles. Madder dyer components were detected in two textile samples, whereas orchil was found in one sample.

The findings show that interdisciplinary multi-analytical research on components of historic fabrics not only expands knowledge about the specific set of textiles analyzed, but also about old manufacturing techniques, allowing for the verification and confirmation of existing hypotheses.

## Figures and Tables

**Table 1:** Description of the grave clothing under investigation.

Sample Code and Image		Item	Description
Sig1	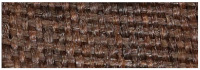	The doublet lining	Silk woven fabric—a taffeta
Sig2	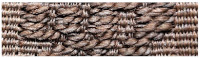	Main fabric of the doublet	Fabric woven with warp, main weft, and supplementary brocading weft, all of silk
Sig3	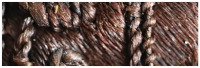	Doublet—embroidered trimming	Silk satin embroidered with silk
Sig4	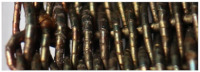	The dalmatic—galloon 1	Plain galloon woven with metal-wrapped thread as a warp and weft
Sig5	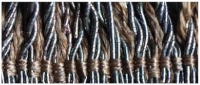	The dalmatic—galloon 2	Patterned galloon woven with silk and metal-wrapped thread
Sig6	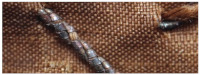	Main fabric of the dalmatic	Silk fabric embroidered with a doubled metal-wrapped thread
Sig7	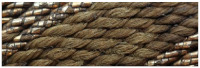	Tassel of the dalmatic	Tassel made of silk and metal wrapped-threads
Sig8	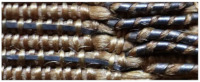	The cope (royal robe)	Fabric woven with warp and main weft of silk. Supplementary wefts: patterning weft of flat metal thread, brocading weft of a metal-wrapped thread
Sig9	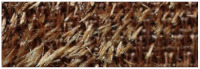	Coffin upholstery	Solid silk velvet
Sig10	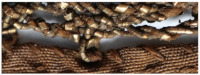	Alb with lace trimming	Alb fabric made of silk warp and weft; Trimming—a bobbin lace of a metal-wrapped thread
Sig11	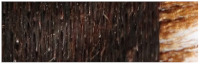	Cassock	Silk satin fabric
Con12	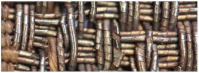	Trimming of the opening and bottom edges of the robe	Patterned galloon woven with warp and weft of metal-wrapped thread
Con13	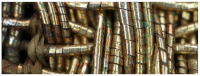	Trimming of the robe wings	Galloon woven with warp and weft of metal-wrapped thread
Con14	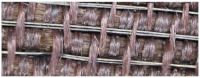	Robe—main fabric	Fabric woven with silk warp and main weft and a metal filament as a patterning weft
Con15	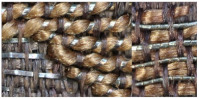	The lining of the robe	Patterned fabric woven with warp and main weft of silk. Supplementary wefts: flat metal thread and metal-wrapped thread as patterning wefts, and metal-wrapped thread as a brocading weft
Con16	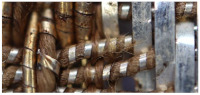	Trimming of the skirt	Patterned galloon woven with two different metal-wrapped threads as weft and main warp and flat, metal thread as a patterning warp
Con17	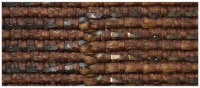	Skirt—main fabric	Fabric woven with silk warp and main weft and two flat metal threads as a patterning wefts
Con18	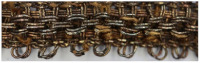	Trimming of the waistcoat	Galloon woven warp and weft made of metal-wrapped thread
Con19	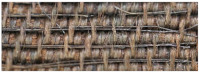	Waistcoat outer fabric	Fabric woven with warp and main weft made of silk. Supplementary patterning weft made of metal filament (wire)
Con20	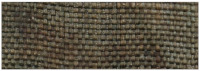	Waistcoat lining	Silk woven fabric—a taffeta
Con21	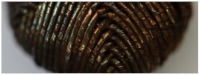	Waistcoat—a button	Needlework made of metal-wrapped thread
Con22	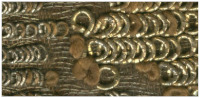	Pillow	Fabric woven with main and binding warps and main weft made of silk, patterned with metal filament, and with two different metal wrapped threads with boucle effect

**Table 2:** Structural parameters and elemental composition of metal threads.

Sample Code	Sample Element	Description	Cu	Ag	Au	w_s_ (mm)	t_s_ (μm)	d_t_ (mm)	Wrap (cm) Direction	Material and Technique
			(at %)							
Sig4	warp and weft	metal-wrapped thread	31.0	52.8	16.2	0.48	13.7	0.33	18; S	silver gilt, beaten and cut
Sig5	weft	metal-wrapped thread	14.2	77.5	8.3	0.33	13.0	0.35	23; S	silver gilt, drawn and rolled
Sig6	embroidery thread	metal-wrapped thread, plied	5.2	65.0	29.9	0.41	16.1	0.39	20; S	silver gilt, drawn and rolled
Sig7	thread	metal-wrapped thread	3.9	70.9	25.2	0.32	16.0	0.29	19; S	silver gilt, drawn and rolled
Sig8	brocading weft	metal-wrapped thread	0.6	90.0	9.4	0.35	13.1	0.46	22; S	silver gilt, drawn and rolled
	patterning weft	flat metal thread	1.1	98.7	0.2	0.15	13.3	-	-	silver gilt, drawn and rolled
Sig10	lace thread	metal-wrapped thread	4.3	78.7	19.7	0.49	13.2	0.48	16; S	silver gilt, drawn and rolled
Con12	wide gallon	metal-wrapped thread	5.2	75.5	19.3	0.38	10.0	0.34	25; S	silver gilt, beaten and cut
Con13	narrow gallon warp and weft	metal-wrapped thread	23.8	54.9	21.3	0.29	10.5	0.45	28; S	silver gilt, beaten and cut
Con14	patterning weft	metal filament	2.7	86.4	10.9	0.06	-	0.06	-	silver gilt, drawn and rolled
Con15	patterning weft 1	flat metal thread	1.1	98.9	0	0.12	11.1	-	-	silver gilt, drawn and rolled
	patterning weft 2	metal-wrapped thread	15.7	66.2	18.1	0.22	12.9	0.17	22; S	silver gilt, beaten and cut
	brocading weft	metal-wrapped thread	40.3	59.7	0	0.12	11.6	0.44	11; S	silver gilt, drawn and rolled
Con16	weft	metal-wrapped thread	61.2	38.8	0	0.25	8.9	0.54	8; S	silver gilt, drawn and rolled
	main warp	metal-wrapped thread	22.0	65.4	12.6	0.53	12.0	0.55	17; S	silver gilt, drawn and rolled
	flushing warp	flat metal thread	2.1	97.9	0	0.65	25.0	-	-	silver gilt, drawn and rolled
Con17	patterning weft 1	flat metal thread	0	85.2	14.8	0.17	9.4	-	-	silver gilt, drawn and rolled
	patterning weft 2	flat metal thread	0	100	0	0.16	8.2	-	-	silver gilt, drawn and rolled
Con18	warp and weft	metal-wrapped thread	24.5	61.0	14.5	0.31	11.3	0.33	30; S	silver gilt, drawn and rolled
Con19	patterning weft	metal filament	0.8	67.1	32.1	0.05	-	-	-	silver gilt, drawn and rolled
Con21	thread	metal-wrapped thread	3.8	72.5	23.7	0.28	13.1	0.29	24; S	silver gilt, beaten and cut
Con22	patterning weft	metal filament	0.6	93.1	6.3	0.07	-	-	-	silver gilt, drawn and rolled
	boucle weft 1	metal-wrapped thread	5.8	79.5	14.7	0.52	13.5	0.65	18; S	silver gilt, drawn and rolled
	boucle weft 2	metal-wrapped thread	3.2	9.8	0	0.40	11.6	0.36	22; S	silver gilt, beaten and cut

w_s_—strip (filament) width; t_s_—strip thickness; d_t_—thread diameter; S—counter-clockwise wrapping direction.

**Table 3:** Colorants identified in royal textiles, possible dyestuffs sources and composition of elements in silk threads.

Sample Code	Identified Compounds	Biological Source	Elements (EDS) * of Silk Threads
Sig1	ellagic acid	tannin plants	Fe, Ca, P, Al, Si, K
Sig2	flavokermesic acid 2-*C*-glucoside (dcII), carminic acid, kermesic acid 7-*C*-glucofuranoside (dcIV), kermesic acid 7-*C*-glucopyranoside (dcVII), ellagic acid	cochineal, tannin plants	Ca, Fe, P, S, Cu, K
Sig3	ellagic acid, carminic acid, flavokermesic acid 2-*C*-glucoside (dcII), kermesic acid 7-*C*-glucofuranoside (dcIV), kermesic acid 7-*C*-glucopyranoside (dcVII)	tannin plants, cochineal	Ca, Fe, P, S, K
Sig4	ellagic acid, carminic acid (traces)	tannin plants, cochineal	Ca, Cu, P, S, Cl, Ag
Sig5	ellagic acid, carminic acid (traces)	tannin plants, cochineal	Ca, P, S, Al, K
Sig6	ellagic acid, carminic acid (traces)	tannin plants, cochineal	S, P, Ca, K, Al
Sig7	carminic acid (traces)	cochineal	Ca, S, Al, P, Cl
Sig8	not detected	-	S, P, Ca, K
Sig9	carminic acid, ellagic acid	cochineal, tannin plants	Ca, S, K, Al
Sig10	ellagic acid, carminic acid (traces)	tannin plants, cochineal	Ca, P, S, Cl
Sig11	carminic acid, dehydrated carminic acid, kermesic acid 7-*C*-glucofuranoside (dc IV), kermesic acid 7-*C*-glucopyranoside (dc VII), ellagic acid, purpurin	cochineal, tannin plants, madder	Ca, P, Mg, S, Fe, Cu
Con12	not performed	-	Al, Na, Mg, Ca, S, K
Con13	not performed	-	Ca, Al, S, P, K, Cl
Con14	carminic acid, indigotin, α-hydroxyorcein, α-aminoorcein, α-aminoorceimine	cochineal, indigo, orchil	Al, Ca, S, Cl, Cu
Con15	indigotin, alizarin, purpurin	indigo, madder	Ca, S, K, Cl, Cu, Si
Con16	not performed	-	S, P, Al, Ca, Cu
Con17	carminic acid, indigotin, kermesic acid 7-*C*-glucofuranoside (dc IV), kermesic acid 7-*C*-glucopyranoside (dc VII), dehydrated carminic acid	cochineal, indigo	Al, Ca, S, Cl, P, Si, Cu
Con18	carminic acid, kermesic acid 7-*C*-glucofuranoside (dc IV), kermesic acid 7-*C*-glucopyranoside (dc VII), dehydrated carminic acid, indigotin	cochineal, indigo	Ca, Cu, P, S, K, Cl
Con19	indigotin	indigo	S, P, K, Ca
Con20	indigotin	indigo	S, P, Al, K, Ca
Con22	carminic acid, dehydrated carminic acid, ellagic acid	cochineal, tannin plants	Ca, Al, Si, S, P

* Elements in order of decreasing signal intensity, from highest to lowest.

**Table 4:** Spectrochromatographic data of the identified dyes.

Peak No.	t_R_ (min)	[M−H]^−^ (*m*/*z*)	[M+H]^+^ (*m*/*z*)	Fragment Ions (*m*/*z*)	Proposed Identification
1	10.2	475	-	431, 341, 282	flavokermesic acid 2-*C*-glucoside (dcII)
2	10.3	491	-	447, 473, 357, 327, 299, 285	carminic acid
3	11.8	491	-	447, 357, 327, 299	kermesic acid 7-*C*-glucofuranoside (dcIV)
4	12.4	491	-	447, 357, 327, 299	kermesic acid 7-*C*-glucopyranoside (dcVII)
5	14.9	313	-	285, 269, 241	flavokermesic acid
6	12.9	473	-	429, 401, 339, 309, 285, 243	dehydrated carminic acid
7	12.9	563	-	269, 252	lucidin *O*-primeveroside
8	12.9	533	-	239	alizarin *O*-primeveroside (ruberythric acid)
9	15.0	239	-	211, 195	hystazarin
10	16.9	239	-	211, 183, 167, 151	alizarin
11	18.4	255	-	227, 183, 171, 129	purpurin
12	20.5	267	-	239, 211, 195	nordamnacanthal
13	2.7	169	-	125, 107, 79	gallic acid
14	8.7	321	-	183, 169, 155, 140, 139, 124	*p*-galloylgallate
15	9.6	787	-	635, 465, 317, 241, 169, 125	tetragalloyl-glucose
16	10.7	939	-	787, 617, 433, 335, 183, 169	pentagalloyl-glucose
17	11.3	301	-	229, 185, 169, 139	ellagic acid
18	17.9	261	-	233, 217, 175	indigotin
19	19.0	261	-	233, 217, 175	indirubin
20	14.3	-	362	347, 346, 345, 331, 278	α-aminoorceimine
21	15.0	-	363	348, 347, 346, 303, 240	α-aminoorcein
22	19.1	-	364	349, 348, 347, 334, 250	α-hydroxyorcein

## Data Availability

Data are included in article/referenced in article.

## Supplementary Materials

The following supporting information can be downloaded at: https://www.mdpi.com/article/10.3390/molecules29010192/s1, Figure S1: EDS spectra of samples presented in Figure 4; Figure S2: Destruction of metal threads; Figure S3: Photograph of the oil painting “Sigismund III Vasa on catafalque”, Christian Melich, 1633; Figure S4: Thermograms of selected thread samples; Table S1: Elemental composition of samples presented in Figure 4 and Figure S1.

## References

[1] Orska-Gawryś J., Surowiec I., Kehl J., Rejniak H., Urbaniak-Walczak K., Trojanowicz M. (2003). Identification of natural dyes in archeological Coptic textiles by liquid chromatography with diode array detection. J. Chromatogr. A.

[2] Blanc R., Espejo T., López-Montes A., Torres D., Crovetto G., Navalón A., Vílchez J.L. (2006). Sampling and identification of natural dyes in historical maps and drawings by liquid chromatography with diode-array detection. J. Chromatogr. A.

[3] Deveoglu O., Torgan E., Karadag R. (2012). Identification by RP-HPLC-DAD of natural dyestuffs from lake pigments prepared with a mixture of weld and dyer’s oak dye plants. J. Liq. Chromatogr. Relat. Technol..

[4] Tamburini D. (2019). Investigating Asian colourants in Chinese textiles from Dunhuang (7th–10th century AD) by high performance liquid chromatography tandem mass spectrometry—Towards the creation of a mass spectra database. Dyes Pigments.

[5] Lech K., Witkos K., Jarosz M. (2014). HPLC-UV-ESI MS/MS identification of the color constituents of sawwort (*Serratula tinctoria* L.). Anal. Bioanal. Chem..

[6] Lech K., Witkos K., Wilenska B., Jarosz M. (2015). Identification of unknown colorants in pre-Columbian textiles dyed with American cochineal (*Dactylopius coccus* Costa) using high-performance liquid chromatography and tandem mass spectrometry. Anal. Bioanal. Chem..

[7] Lech K., Jarosz M. (2016). Identification of Polish cochineal (*Porphyrophora polonica* L.) in historical textiles by high-performance liquid chromatography coupled with spectrophotometric and tandem mass spectrometric detection. Anal. Bioanal. Chem..

[8] Lech K. (2020). Universal analytical method for characterization of yellow and related natural dyes in liturgical vestments from Krakow. J. Cult. Herit..

[9] Lech K., Fornal E. (2020). A Mass Spectrometry-Based Approach for Characterization of Red, Blue, and Purple Natural Dyes. Molecules.

[10] Tamburini D., Dyer J., Davit P., Aceto M., Turina V., Borla M., Vandenbeusch M., Gulmini M. (2019). Compositional and Micro-Morphological Characterisation of Red Colourants in Archaeological Textiles from Pharaonic Egypt. Molecules.

[11] Petroviciu I., Teodorescu I., Albu F., Virgolici M., Nagoda E., Medvedovici A. (2019). Dyes and biological sources in nineteenth to twentieth century ethnographic textiles from Transylvania, Romania. Herit. Sci..

[12] Otłowska O., Ślebioda M., Kot-Wasik A., Karczewski J., Śliwka-Kaszyńska M. (2018). Chromatographic and spectroscopic identification and recognition of natural dyes, uncommon dyestuff components and mordants in 16th century carpet. Molecules.

[13] Degano I., Tognotti P., Kunzelman D., Modugno F. (2017). HPLC-DAD and HPLC-ESI-Q-ToF characterisation of early 20th century lake and organic pigments from Lefranc archives. Herit. Sci..

[14] Śliwka-Kaszyńska M., Ślebioda M., Brillowska-Dąbrowska A., Mroczyńska M., Karczewski J., Marzec A., Rybiński P., Drążkowska A. (2021). Multi-Technique Investigation of Grave Robes from 17th and 18th Century Crypts Using Combined Spectroscopic, Spectrometric Techniques, and New-Generation Sequencing. Materials.

[15] Degano I., Biesaga M., Colombini M.P., Trojanowicz M. (2011). Historical and archaeological textiles: An insight on degradation products of wool and silk yarns. J. Chromatogr. A.

[16] Surowiec I., Szostek B., Trojanowicz M. (2007). HPLC-MS of anthraquinoids, flavonoids, and their degradation products in analysis of natural dyes in archeological objects. J. Sep. Sci..

[17] Sabatini F., Lluveras-Tenorio A., Degano I., Kuckova S., Krizova I., Colombini M.P. (2016). A matrix-assisted laser desorption/ionization time-of-flight mass spectrometry method for the identification of anthraquinones: The case of historical lakes. J. Am. Soc. Mass Spectrom..

[18] Kramell A.E., García-Altares M., Pötsch M., Kluge R., Rother A., Hause G., Hertweck C., Csuk R. (2019). Mapping Natural Dyes in Archeological Textiles by Imaging Mass Spectrometry. Sci. Rep..

[19] Selvius DeRoo C.S., Armitage R.A. (2011). Direct Identification of Dyes in Textiles by Direct Analysis in Real Time—Time of Flight Mass Spectrometry. Anal. Chem..

[20] Tamburini D., Cartwright C.R., Pullan M., Vickers H. (2018). An investigation of the dye palette in Chinese silk embroidery from Dunhuang (Tang dynasty). Archaeol. Anthropol. Sci..

[21] Chen V.J., Smith G.D., Holden A., Paydar N., Kiefer K. (2016). Chemical analysis of dyes on an Uzbek ceremonial coat: Objective evidence for artifact dating and the chemistry of early synthetic dyes. Dyes Pigments.

[22] Degano I., Mattonai M., Sabatini F., Colombini M.P. (2019). A mass spectrometric study on tannin degradation within dyed woolen yarns. Molecules.

[23] Nabais P., Oliveira J., Pina F., Teixeira N., Freitas V., Bras N.F., Clemente A., Rangel M., Silva A.M.S., Melo M.J. (2020). A 1000 year-old mystery solved: Unlocking the molecular structure for the medieval blue from *Chrozophora tinctoria*, also known as folium. Sci. Adv..

[24] Tamburini D., Breitung E., Mori C., Kotajima T., Clarke M.L., McCarthy B. (2020). Exploring the transition from natural to synthetic dyes in the production of 19th-century Central Asian ikat textiles. Herit. Sci..

[25] Tamburini D., Dyer J., Cartwright C., Green A. (2023). Changes in the production materials of Burmese textiles in the nineteenth century-dyes, mordants and fibres of Karen garments from the British Museum’s collection. Herit. Sci..

[26] Otłowska O., Ślebioda M., Wachowiak M., Śliwka-Kaszyńska M. (2015). Identification and characterization of the Indian Yellow dyestuff and its degradation products in historical oil paint tube by liquid chromatography mass spectrometry. RSC Adv..

[27] Otłowska O., Ślebioda M., Wachowiak M., Śliwka-Kaszyńska M. (2017). A multi-analytical approach to the characterization of natural organic dyestuffs and inorganic substrates present in the 19th-century artistic oil paints manufactured by a French art materials supplier Richard Aines. Anal. Methods.

[28] Deyjoo R., Holakooei P., Sabatini F., Degano I., Colombini M.P. (2021). Coptic textiles in Tehran: Dye and fibre characterisation in four Coptic textiles preserved at the Moghadam Museum. Archaeol. Anthropol. Sci..

[29] Lech K., Nawała J., Popiel S. (2021). Mass spectrometry for investigation of natural dyes in historical textiles: Unveiling the mystery behind safflower-dyed fibers. J. Am. Soc. Mass Spectrom..

[30] Nakamura R., Tanaka Y., Ogata A., Naruse M. (2009). Dye analysis of shosoin textiles using excitation−emission matrix fluorescence and ultraviolet-visible reflectance spectroscopic techniques. Anal. Chem..

[31] Aceto M., Agostino A., Fenoglio G., Idone A., Gulmini M., Picollo M., Ricciardi P., Delaney J.K. (2014). Characterisation of colourants on illuminated manuscripts by portable fibre optic UV-visible-NIR reflectance spectrophotometry. Anal. Methods.

[32] Maynez-Rojas M.A., Casanova-González E., Ruvalcaba-Sil J.L. (2017). Identification of natural red and purple dyes on textiles by Fiber-optics Reflectance Spectroscopy. Spectrochim. Acta Part A Mol. Biomol. Spectrosc..

[33] Mounier A., Le Bourdon G., Aupetit C., Lazare S., Biron C., Pérez-Arantegui J., Almazán D., Aramendia J., Prieto-Taboada N., Fdez-Ortiz de Vallejuelo S. (2018). Red and blue colours on 18th–19th century Japanese woodblock prints: In situ analyses by spectrofluorimetry and complementary non-invasive spectroscopic methods. Microchem. J..

[34] Villafana T., Edwards G. (2019). Creation and reference characterization of Edo period Japanese woodblock printing ink colorant samples using multimodal imaging and reflectance spectroscopy. Herit. Sci..

[35] Dyer J., Tamburini D., O’Connell E.R., Harrison A. (2018). A multispectral imaging approach integrated into the study of Late Antique textiles from Egypt. PLoS ONE.

[36] de Ferri L., Tripodi R., Martignon A., Ferrari E.S., Lagrutta-Diaz A.C., Vallotto D., Pojana G. (2018). Non-invasive study of natural dyes on historical textiles from the collection of Michelangelo Guggenheim. Spectrochim. Acta Part A Mol. Biomol. Spectrosc..

[37] Hložek M., Trojek T., Prokeš R., Linhart V. (2019). Mediaeval metal threads and their identification using micro-XRF scanning, confocal XRF, and X-ray micro-radiography. Radiat. Phys. Chem..

[38] Enguita O., Climent-Font A., Garcia G., Montero I., Fedi M.E., Chiari M., Lucarelli F. (2002). Characterization of metal threads using different PIXE analysis. Nucl. Instrum. Methods Phys. Res. Sect. B Beam Interact. Mater. At..

[39] Simic K., Zamboni I., Fazinic S., Mudronja D., Sovic L., Gouasmia S., Soljacic I. (2018). Comparative analysis of textile metal threads from liturgical vestments and folk costumes in Croatia. Nucl. Instrum. Methods Phys. Res. Sect. B Beam Interact. Mater. At..

[40] Duran A., Perez-Maqueda R., Perez-Rodriguez J.L. (2019). Degradation processes of historic metal threads used in some Spanish and Portuguese ornamentation pieces. J. Cult. Herit..

[41] Shibayama N., Wypyski M., Gagliardi-Mangilli E. (2015). Analysis of natural dyes and metal threads used in 16th–18th century Persian/Safavid and Indian/Mughal velvets by HPLC-PDA and SEM-EDS to investigate the system to differentiate velvets of these two cultures. Herit. Sci..

[42] Simic K., Soljacic I., Mudronja D., Petrovic Les T. (2022). Metal Content and Structure of Textiles in Textile Metal Threads in Croatia from 17th to 20th Century. Materials.

[43] Ferreira F., Moreiras H., Manhita A., Tomaz P., Mirao J., Dias C.B., Caldeira A.T. (2015). The Liturgical Cope of D. Teotónio of Braganza: Material Characterization of a 16th Century Pluviale, Microsc. Microanal..

[44] Cybulska M., Jedraszek-Bomba A., Kuberski S., Wrzosek H. (2008). Methods of Chemical and Physicochemical Analysis in the Identification of Archaeological and Historical Textiles. Fibres Text. East. Eur..

[45] Cybulska M., Seiko J., Sabu T., Pintu P., Ritu P. (2003). Analysis and Visualization of Historical Textiles for the Needs of Museum Conservation and Exhibition. Handbook of Museum Textiles: Volume II Scientific and Technological Research.

[46] Járó M., Tóth A.L. (1991). Scientific identification of European metal thread manufacturing techniques of the 17–19th centuries. Endeavour.

[47] Járó M., Gál T., Tóth A. (2000). The Characterization and Deterioration of Modern Metallic Threads. Stud. Conserv..

[48] Karatzani A., Tzachili I., Zimi E. (2012). Metal Threads: The Historical Development. Textiles and Dress in Greece and the Roman East: A Technological and Social Approach.

[49] Crowfoot E., Pritchard F., Staniland K. (2006). Textiles and Clothing, C.1150–C.1450.

[50] Cybulska M., Kuberski S., Maik J., Orlińska-Mianowska E., Neckar E., Banck-Burgess J., Nübold C. (2013). Figural embroidery from Tum Collegiate Church–analysis, reconstruction and identification. NESAT XI, Proceedings of the North European Symposium for Archaeological Textiles XI, Esslingen, Germany, 10–13 May 2011.

[51] Rogerson C., Garside P., Lennard F., Hayward M. (2005). Instrumental analysis of metal threads as an aid for interpretation and preservation of a fifteenth-century tapestry altar frontal and super frontal. Tapestry Conservation: Principles and Practice.

[52] Bonito Fanelli R. (1981). Five Centuries of Italian Textiles 1300–1800. A Selection from the Museo del Tessuto Prato.

[53] Cybulska M., Maik J. (2007). Archaeological Textiles: A Need for New Methods of Analysis and Reconstruction. Fibres Text. East. Eur..

[54] Indictor N., Ballard M.W. The effects of aging on textiles that contain metal: Implications for analyses. Proceedings of the Conservation of Metals: Problems in the Treatment of Metal-Organic and Metal-Inorganic Composite Objects: International Restorer Seminar.

[55] Hofenk de Graaff J.H. (2004). The Colourful Past. Origins Chemistry and Identification of Natural Dyestuffs.

[56] Cardon D. (2007). Natural Dyes: Sources, Tradition, Technology and Science.

[57] Perez-Rodriguez J.L., Perez-Maqueda R., Luisa Franquelo M., Duran A. (2018). Study of the thermal decomposition of historical metal threads. J. Therm. Anal. Calorim..

[58] Gupta P., Kumar M., Bhardwaj N., Kumar J.P., Krishnamurthy C.S., Nandi S.K., Mandal B.B. (2016). Mimicking Form and Function of Native Small Diameter Vascular Conduits Using Mulberry and Non-mulberry Patterned Silk Films. ACS Appl. Mater. Interfaces.

[59] Lee J., Kim M., Lee K., van Elslande E., Walterband P., Lee Y. (2014). Analysis of natural dyes in archeological textilesusing TOF-SIMS and other analytical techniques. Surf. Interface Anal..

[60] Marcela Sepúlveda M., Lemp Urzúa C., Cárcamo-Vega J., Casanova-Gónzalez E., Gutiérrez S., Maynez-Rojas M.A., Ballester B., Ruvalcaba-Sil J.L. (2021). Colors and dyes of archaeological textiles from Tarapacá in the Atacama Desert (South Central Andes). Herit. Sci..

[61] Melo M.J., Bechtold T., Mussak R. (2009). History of Natural Dyes in the Ancient Mediterranean World, in Handbook of Natural Colorants.

[62] Cuyckens F., Claeys M. (2004). Mass spectrometry in the structural analysis of flavonoids. J. Mass Spectrom..

[63] Ferreira E.S.B., Hulme A.M., McNaby H., Quye A. (2004). The natural constituents of historical textile dyes. Chem. Soc. Rev..

