# Supplementary material for: Multi-Analytical Techniques for the Study of Burial Clothes of Polish King Sigismund III Vasa (1566–1633) and His Wife Constance Habsburg (1588–1631)
Source: Molecules. 2023 Dec 28;29(1):192. doi: 10.3390/molecules29010192 (PMC10780732; doi:10.3390/molecules29010192)
Supplement: Supplementary file 1 [file molecules-29-00192-s001.zip › molecules-2743989-supplementary.pdf]

Supplementary Materials

Multi-Analytical Techniques for the Study of Burial Clothes of Polish King Sigismund III Vasa (1566–1633) and His Wife Constance Habsburg (1588–1631)

Magdalena Śliwka-Kaszyńska [1,*], Maria Cybulska [2], Anna Drążkowska [3], Sławomir Kuberski [4], Jakub Karczewski [5], Anna Marzec [6] and Przemysław Rybiński [7]

[1] Department of Organic Chemistry, Faculty of Chemistry, Gdansk University of Technology, 80-233 Gdańsk, Poland
[2] Faculty of Material Technologies and Textile Design, Institute of Architecture of Textiles, Lodz University of Technology, 90-924 Lodz, Poland; maria.cybulska@p.lodz.pl
[3] Faculty of History, Institute of Archaeology, Nicolaus Copernicus University in Torun, 87-100 Torun, Poland; annadr@umk.pl
[4] Faculty of Process and Environmental Engineering, Lodz University of Technology, 93-005 Lodz, Poland; slawomir.kuberski@p.lodz.pl
[5] Faculty of Applied Physics and Mathematics, Gdansk University of Technology, 80-233 Gdańsk, Poland; jakkarcz@pg.edu.pl
[6] Faculty of Chemistry, Lodz University of Technology, 90-924 Lodz, Poland; anna.marzec@p.lodz.pl
[7] Institute of Chemistry, Jan Kochanowski University, 25-369 Kielce, Poland; przemyslaw.rybinski@ujk.edu.pl
* Correspondence: magkaszy@pg.edu.pl; Tel.: +48-58-347-1942

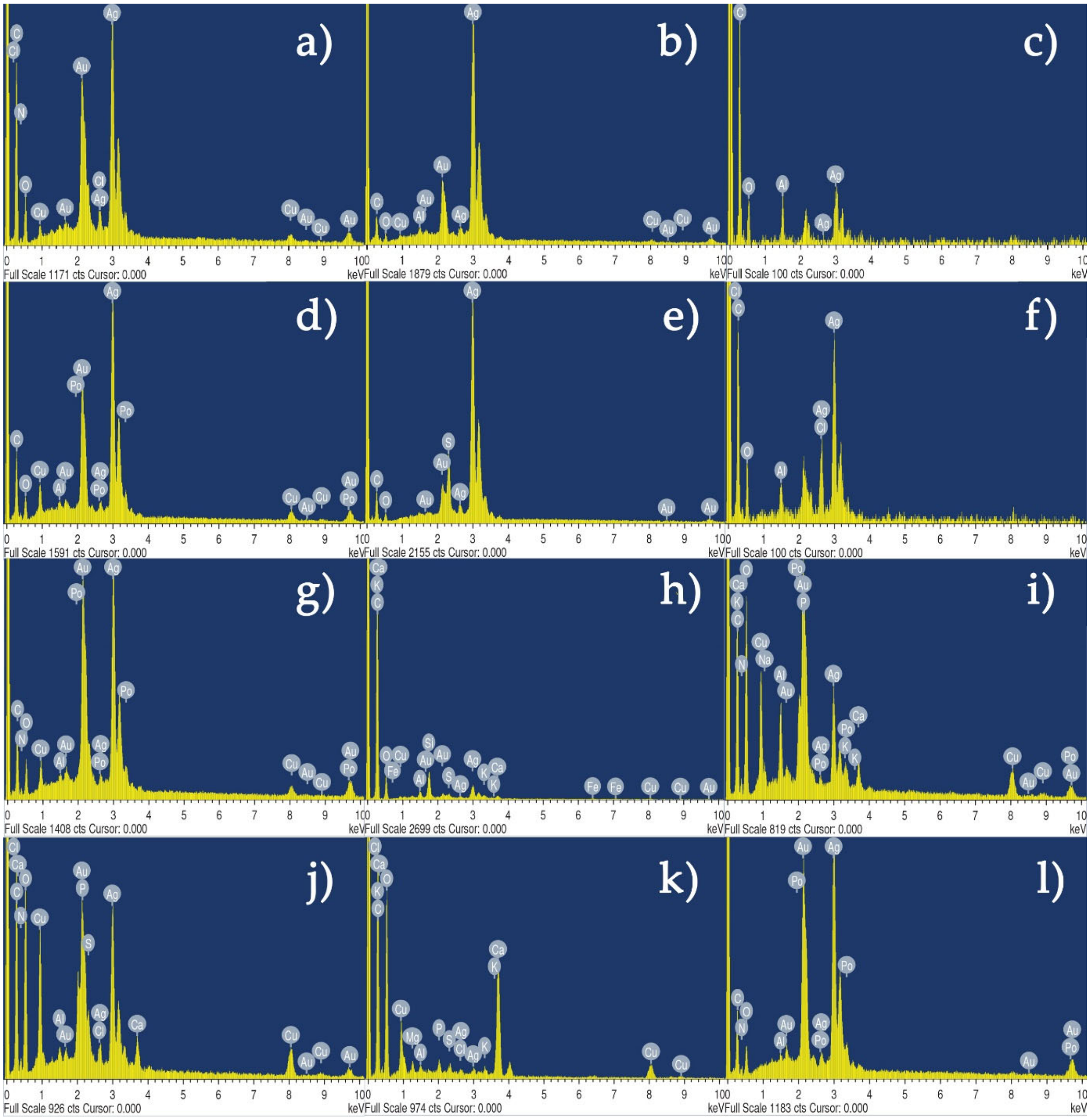

Figure S1. EDS spectra of samples presented in Figure 4: (**a**) Sig10, (**b**) Con19, (**c**) Con21, (**d**) Con16, (**e**) Sig8, (**f**) Sig4, (**g**) Sig7, (**h**) Con22, (**i**) Con13, (**j**) Sig6, (**k**) Con18, and (**l**) Con19.

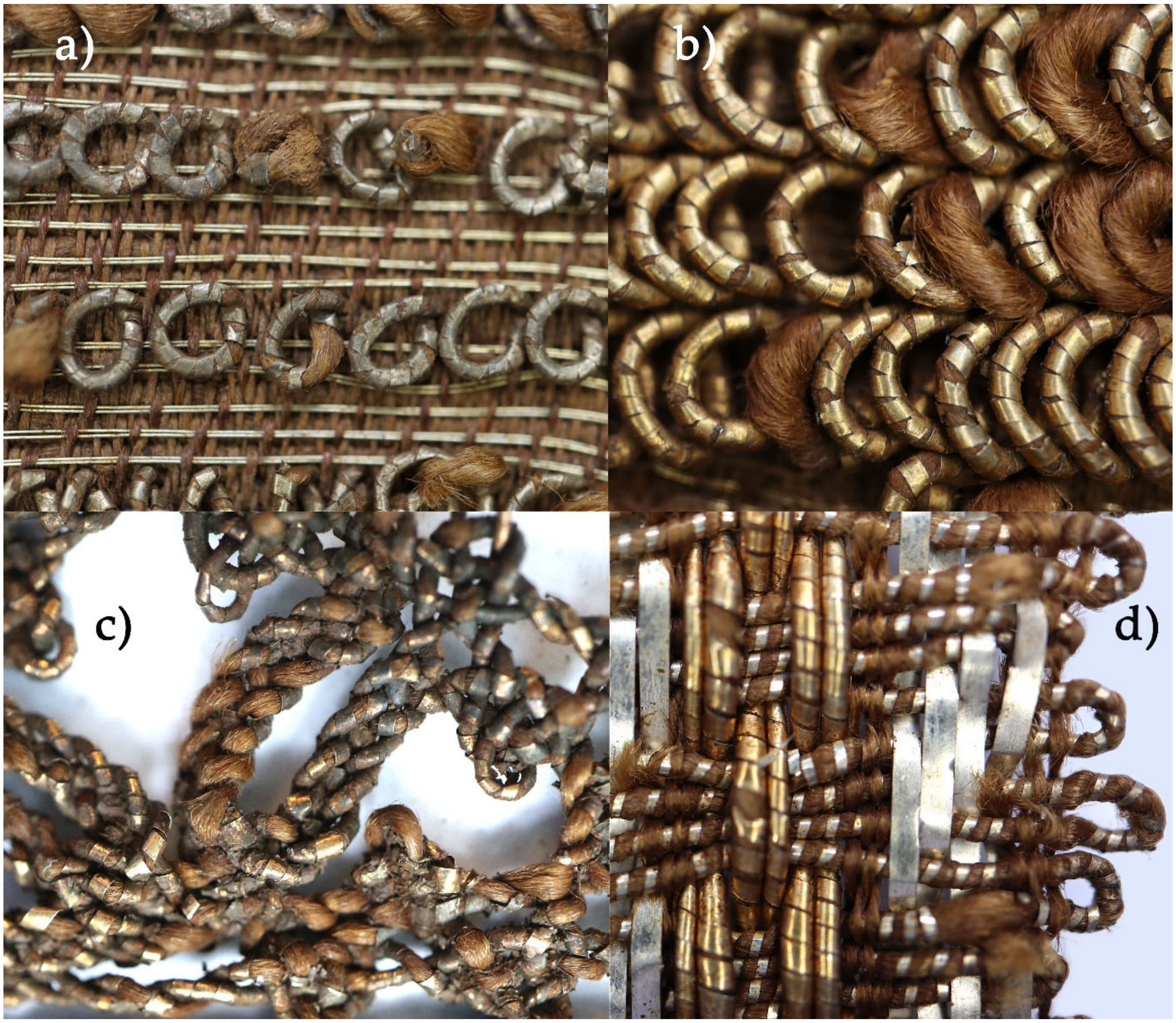

Figure S2. Destruction of metal threads: (**a**,**b**) fabric with boucle effect (Con22); (**c**) lace (Sig10); (**d**) woven tape (Con16), photos by M. Cybulska.

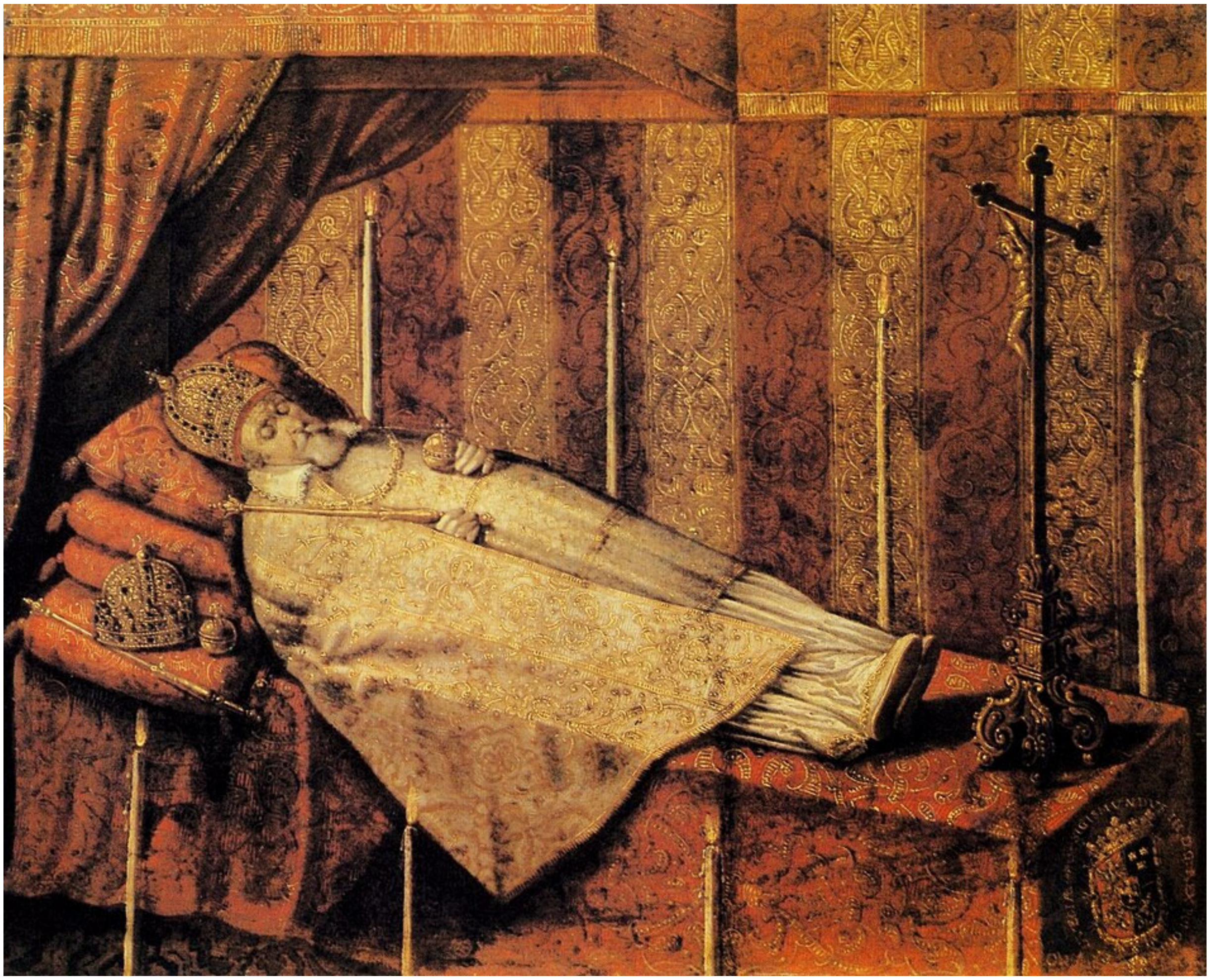

Figure S3. Christian Melich, Sigismund III Vasa on catafalque, 1633, dep. Wawel Castle. Source: https://pl.wikipedia.org/wiki/Plik:Melich_Sigismund_III_Vasa_on_catafalque.jpg

(**a**)

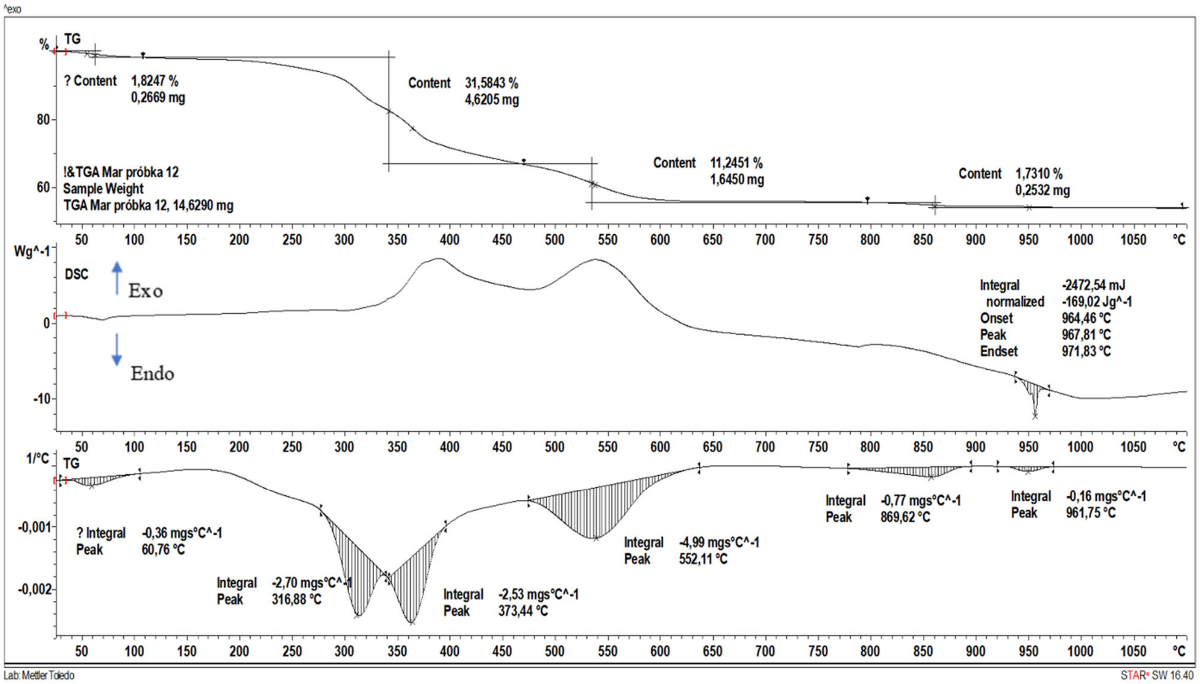
^exo
TG
%
? Content 1,8247 %
0,2669 mg
Content 31,5843 %
4,6205 mg
Content 11,2451 %
1,6450 mg
Content 1,7310 %
0,2532 mg
!&TGA Mar próbka 12
Sample Weight
TGA Mar próbka 12, 14,6290 mg
80
60
50 100 150 200 250 300 350 400 450 500 550 600 650 700 750 800 850 900 950 1000 1050 °C
Wg^-1
DSC
Exo
Endo
0
-10
Integral -2472,54 mJ
normalized -169,02 Jg^-1
Onset 964,46 °C
Peak 967,81 °C
Endset 971,83 °C
1/°C
TG
? Integral -0,36 mgs°C^-1
Peak 60,76 °C
Integral -2,70 mgs°C^-1
Peak 316,88 °C
Integral -2,53 mgs°C^-1
Peak 373,44 °C
Integral -4,99 mgs°C^-1
Peak 552,11 °C
Integral -0,77 mgs°C^-1
Peak 869,62 °C
Integral -0,16 mgs°C^-1
Peak 961,75 °C
-0,001
-0,002
Lab: Mettler Toledo
STAR® SW 16.40

(**b**)

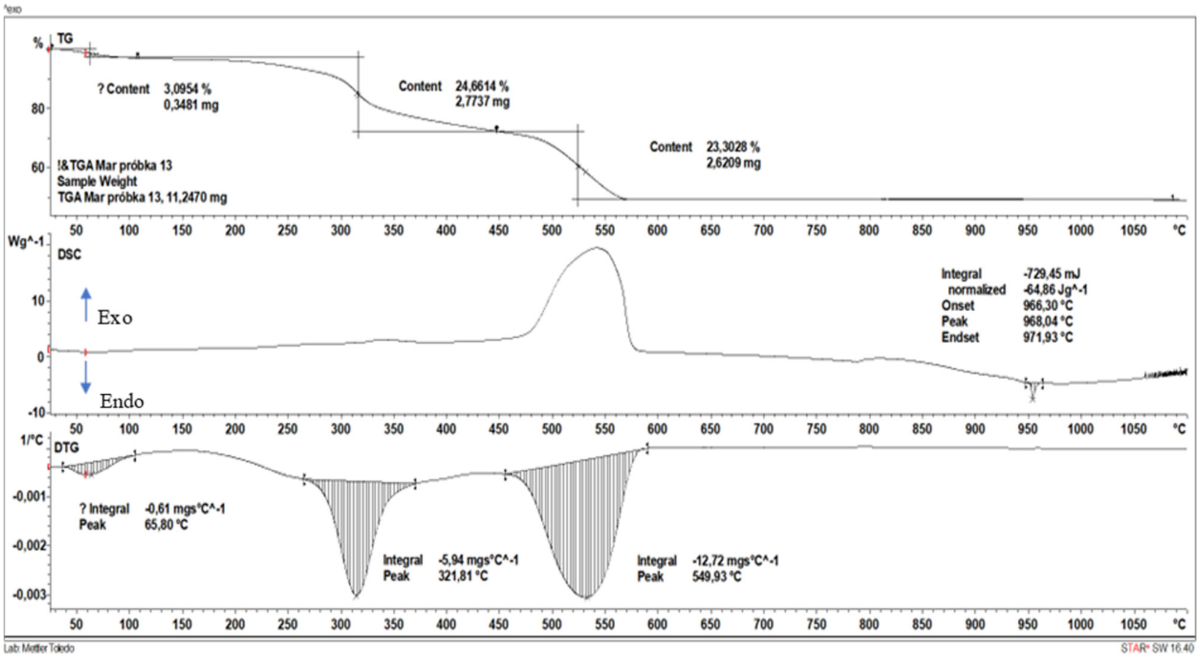
^exo
TG
%
? Content 3,0954 %
0,3481 mg
Content 24,6614 %
2,7737 mg
Content 23,3028 %
2,6209 mg
!&TGA Mar próbka 13
Sample Weight
TGA Mar próbka 13, 11,2470 mg
80
60
50 100 150 200 250 300 350 400 450 500 550 600 650 700 750 800 850 900 950 1000 1050 °C
Wg^-1
DSC
Exo
Endo
10
0
-10
Integral -729,45 mJ
normalized -64,86 Jg^-1
Onset 966,30 °C
Peak 968,04 °C
Endset 971,93 °C
1/°C
DTG
? Integral -0,61 mgs°C^-1
Peak 65,80 °C
Integral -5,94 mgs°C^-1
Peak 321,81 °C
Integral -12,72 mgs°C^-1
Peak 549,93 °C
-0,001
-0,002
-0,003
Lab: Mettler Toledo
STAR® SW 16.40

(**c**)

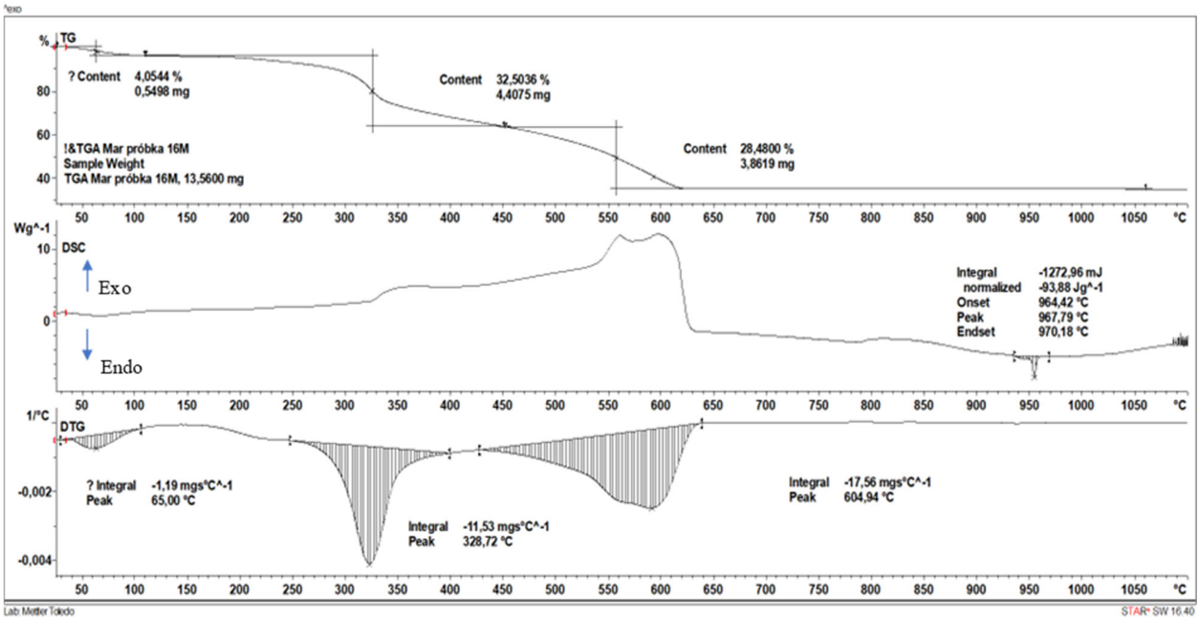

^exo
TG
%
? Content 4,0544 %
0,5498 mg
Content 32,5036 %
4,4075 mg
Content 28,4800 %
3,8619 mg
!&TGA Mar próbka 16M
Sample Weight
TGA Mar próbka 16M, 13,5600 mg
Wg^-1
DSC
Exo
Endo
Integral -1272,96 mJ
normalized -93,88 Jg^-1
Onset 964,42 °C
Peak 967,79 °C
Endset 970,18 °C
1/°C
DTG
? Integral -1,19 mgs°C^-1
Peak 65,00 °C
Integral -11,53 mgs°C^-1
Peak 328,72 °C
Integral -17,56 mgs°C^-1
Peak 604,94 °C
Lab: Mettler Toledo
STARe SW 16.40

(**d**)

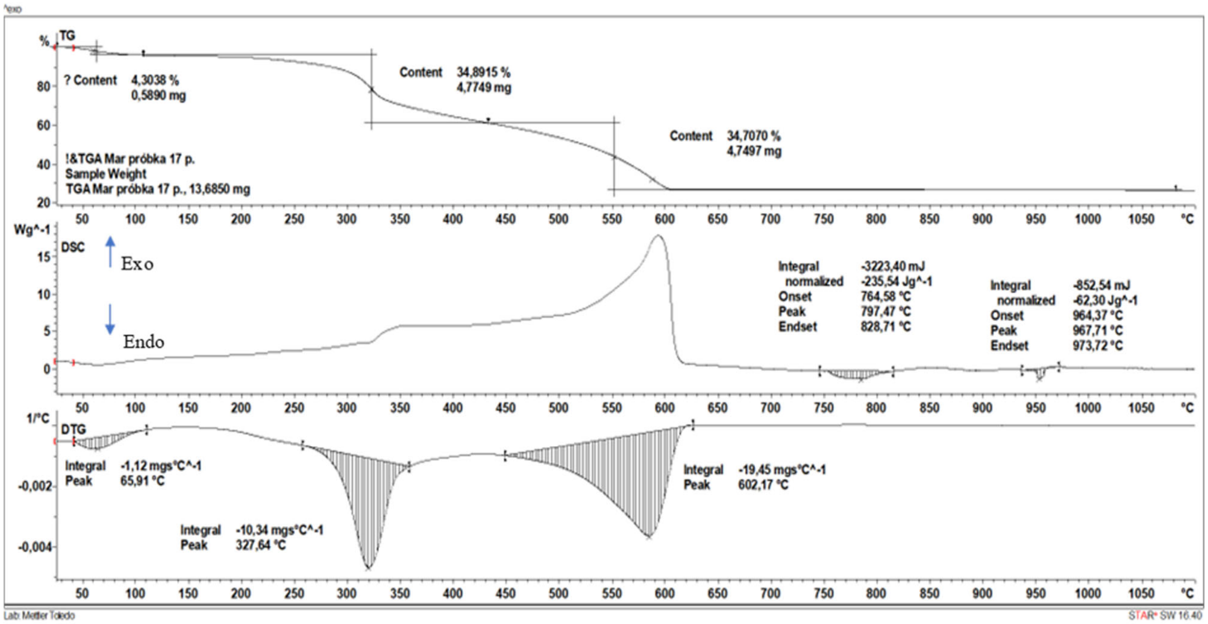

^exo
TG
%
? Content 4,3038 %
0,5890 mg
Content 34,8915 %
4,7749 mg
Content 34,7070 %
4,7497 mg
!&TGA Mar próbka 17 p.
Sample Weight
TGA Mar próbka 17 p., 13,6850 mg
Wg^-1
DSC
Exo
Endo
Integral -3223,40 mJ
normalized -235,54 Jg^-1
Onset 764,58 °C
Peak 797,47 °C
Endset 828,71 °C
Integral -852,54 mJ
normalized -62,30 Jg^-1
Onset 964,37 °C
Peak 967,71 °C
Endset 973,72 °C
1/°C
DTG
Integral -1,12 mgs°C^-1
Peak 65,91 °C
Integral -10,34 mgs°C^-1
Peak 327,64 °C
Integral -19,45 mgs°C^-1
Peak 602,17 °C
Lab: Mettler Toledo
STARe SW 16.40

(**e**)

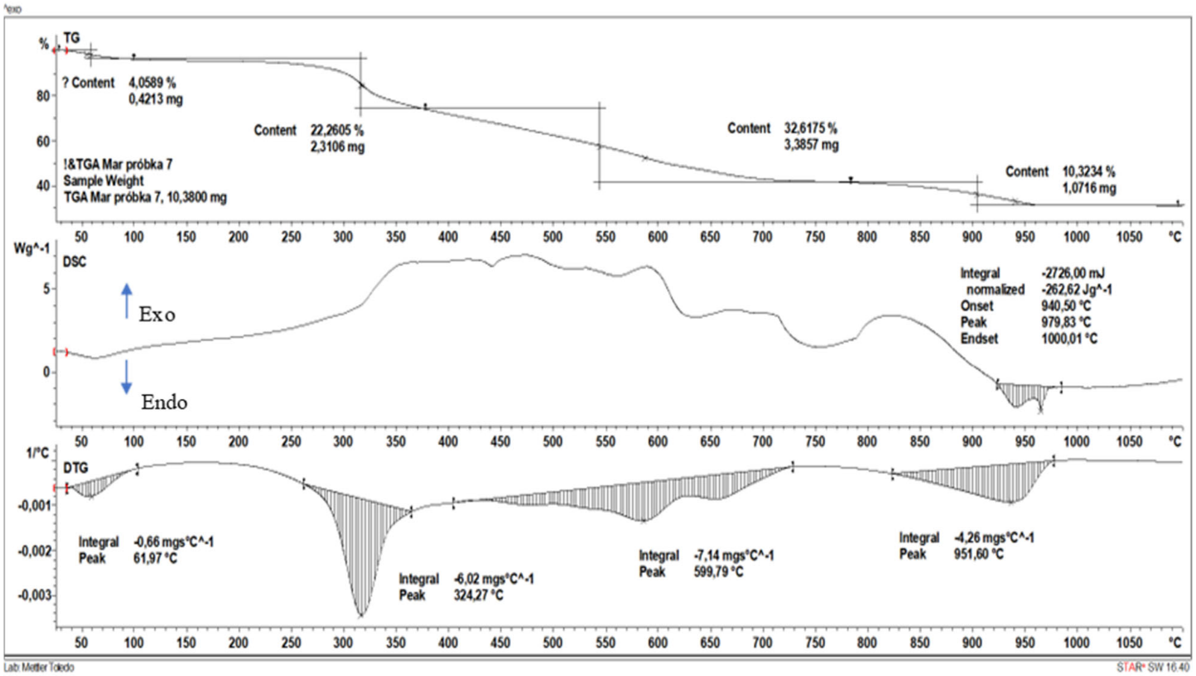

Figure S4. Thermograms for samples: (**a**) Con 12 (**b**) Con 13, (**c**) Con 16, (**d**) Con 17, (**e**) Sig 7.

Table S1. Elemental composition of samples presented in Figures 4 and S1.

Sample	a (Sig10)	b (Con19)	c (Con21)	d Con16	e (Sig8)	f (Sig4)	g (Sig7)	h (Con22)	i (Con13)	j (Sig6)	k (Con18)	l (Con19)
**Elements**							Weight %					
**C**	51.81	8.83	3.36	23.06	15.29	5.24	21.29	117.90	50.35	63.29	59.12	17.02
**N**	11.21	x	x	x	x		9.21		10.22	17.75		9.60
**O**	23.85	7.34	1.91	16.27	9.56	3.15	18.12	29.31	51.03	63.51	58.90	14.09
**Na**									1.21			
**Si**								3.24				
**S**					7.43			0.63		2.86		
**K**								0.47	1.09		0.46	
**Ca**								0.65		2.49	0.49	
**Fe**								0.55				
**P**									3.62	4.42	0.74	
**Ca**									1.88		9.46	
**Mg**											0.70	
**Al**	x	0.66	0.18	0.63	x	0.17	0.54	0.77	3.17	0.67	0.32	0.44
**Cl**	1.53	x	x	x	x	0.48				1.18	0.30	
**Cu**	3.87	1.54	x	5.95	x		4.89	0.55	9.35	11.27	6.41	
**Ag**	55.01	74.20	0.88	68.05	82.01	3.55	57.02	6.31	15.22	27.61	1.01	51.67
**Au**	30.65	19.48	x	37.86	11.72	x	42.97	2.04	31.10	28.32	x	42.43
**Total**	177.92	112.06	6.32	151.82	126.01	12.59	154.04	162.41	178.30	223.37	137.91	135.52
